# Antifungal Structure–Activity Relationship Studies of Broad-Spectrum Phenothiazines

**DOI:** 10.1021/acsomega.4c09833

**Published:** 2025-04-29

**Authors:** Samantha C. Brosend, Soumitra Guin, Gregory Giovine, Carlos Gadalla, Miguel A. Campos, Alisa Mara, Nicholas G. Jentsch, Haresh Thakellapalli, Kathryn M. Alden, Sarah R. Beattie, Damian J. Krysan, Marvin J. Meyers

**Affiliations:** † Department of Chemistry, School of Science and Engineering, Saint Louis University, Saint Louis, Missouri 63103, United States; ‡ Department of Pediatrics, Carver College of Medicine, University of Iowa, Iowa City, Iowa 52242, United States; § Department of Molecular Physiology and Biophysics, University of Iowa, Iowa City, Iowa 52242, United States; ∥ Institute for Drug and Biotherapeutic Innovation, Saint Louis University, St. Louis, Missouri 63103, United States

## Abstract

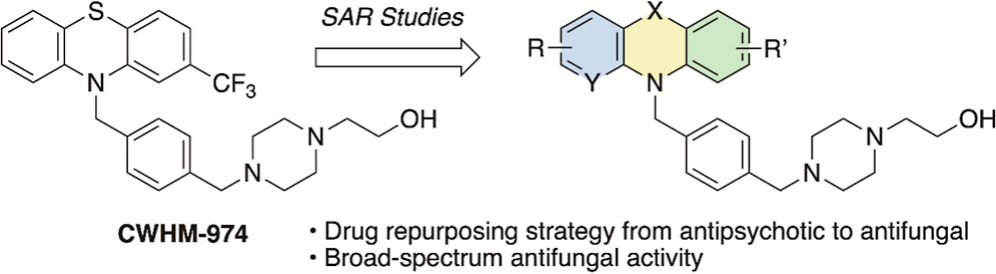

Fungal infections remain a critical unmet medical need with millions of infections occurring annually. With only three classes of antifungal drugs available, drug resistance and modest activity toward some fungi represent threats to human health. To address this, optimization of the antifungal properties of approved drugs with appropriate pharmacokinetic properties represents an attractive strategy. Here, we have shown that the antifungal activity of phenothiazine-based **CWHM-974** extends to include fluconazole-resistant *Candida albicans*, *Candida auris*, and *Cryptococcus glabrata*, filamentous molds such as *Aspergillus fumigatus*, *Fusarium* spp., and *Rhizopus arrhizus*, endemic human fungal pathogens *Histoplasma capsulatum*, *Blastomyces dermatitidis*, and *Coccidioides* spp. Thus, phenothiazines (PTZs) have consistent antifungal activity toward a broad range of medically relevant fungi, including organisms that range from difficult to nearly impossible to treat with current drugs. Unfortunately, **CWHM-974** did not exhibit in vivo efficacy in either *Cryptococcus neoformans* or *C. albicans* mouse infection models, necessitating an effort to optimize the scaffold further. Toward this end, synthesis and minimum inhibitory concentration (MIC) values are reported for 15 novel PTZ analogs to extend structure–activity relationships (SARs). Six analogs were identified as 2- to 4-fold more potent. Azaphenothiazines (aza-PTZs) were tolerated and resulted in potent antifungals with moderate reduction in lipophilicity and more facile chemical synthesis. One analog displayed modest selectivity improvement against the serotonin 5HT_2c_ receptor versus **CWHM-974**, but its overall selectivity profile versus a panel of other serotonin and dopamine receptors did not improve. Overall, the broad-spectrum antifungal activity and reduced neuroreceptor affinity of PTZ-based analogs encourages continued optimization to develop a novel antifungal therapeutic drug.

## Introduction

The development of new classes of antifungal drugs is a critical unmet clinical need.^1^ The global burden of fungal disease is under-appreciated, and it is estimated that millions of infections occur each year. Recently, The World Health Organization (WHO) assembled a Fungal Pathogen Priority List to highlight the health impact of these pathogens and catalyze interest in addressing this clinical need.^2^ These fungal infections affect almost every human organ system and are caused by a genomically and phylogenetically diverse set of fungal pathogens. Unfortunately, we currently have only three classes of antifungal drugs that can be used as a primary therapy for life-threatening fungal infections.^3^ Furthermore, it has been nearly 25 years since a new class of antifungal drugs, the echinocandins, was introduced into clinical use.

To address this critical unmet clinical need, development of novel antifungal treatment regimens is urgently needed. Many groups have explored a variety of approaches,^3−7^ including a repurposing strategy in which libraries of previously approved drugs are screened for antifungal activity.^8^ One goal of a repurposing approach is to identify a drug with antifungal activity at concentrations achievable in human tissues to allow a direct path to clinical trials. To date, only two repurposed drugs have been studied in clinical trials, and neither proved efficacious.^9,10^ Alternatively, a repurposing approach can identify drugs with favorable pharmacology and promising antifungal activity, which can then form the basis for systematic optimization of antifungal activity and minimization of known side effects.

Pursuing the latter goal, we have focused on optimizing the antifungal properties of the PTZ scaffold and minimizing its interactions with neurotransmitter receptors.^11,12^ Our initial studies have focused on derivatives of the antipsychotic medication, fluphenazine. Our interest in the PTZ scaffold was driven in part by its ability to penetrate the central nervous system (CNS). The CNS is an important target organ for multiple fungal infections. For example, *Cryptococcus neoformans* is the second leading cause of infectious disease-related deaths in people living with human immunodeficiency virus/acquired immunodeficiency syndrome (HIV/AIDS), and infection causes meningoencephalitis.^13^ The primary treatment for cryptococcal meningitis is amphotericin B, which is the oldest antifungal drug in the pharmacopeia (introduced in 1957) and is highly toxic. Since this infection causes >100,000 deaths per year, and no new therapies have been developed in 50 years, it is a priority for new antifungal drugs to easily access the CNS.^14^

**Figure 1:**
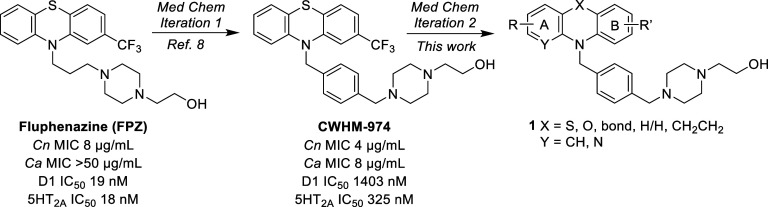
Origin of **CWHM-974** and medicinal chemistry strategy.

Although multiple PTZs have been shown to have antifungal activity, we, and others, have shown that the antipsychotic medication fluphenazine is among those most active of the clinically used PTZs against *C. neoformans* and *Candida* spp.^11^ We have also shown that an analog of fluphenazine (**CWHM-974**) with reduced affinity for neurotransmitter receptors (see Figure 1 and Table 3 below) has improved antifungal activity and leads to less toxicity when tested in mouse models.^12,15^ In addition, these studies have shown that fluphenazine derivatives are active against drug-resistant *Candida* spp., partly because they are not susceptible to efflux pump-mediated resistance mechanisms.^16^

Here, we report on the further evaluation and optimization of antifungal fluphenazine analogs. We show that our initial lead **CWHM-974** has broad-spectrum activity against a wide range of human fungal pathogens but does not yet have sufficient activity or tolerability to achieve in vivo efficacy. As such, we have undertaken additional medicinal chemistry optimization strategies that focus on further reducing neurotransmitter antagonism and improving antifungal activity.

## Results and Discussion

### **CWHM-974** Has Broad-Spectrum Antifungal Activity

As discussed above, while this project was primarily motivated by the goal of developing new drugs to treat cryptococcal meningitis, broad-spectrum agents would be even more valuable. Additionally, the brain is a secondary target organ for other fungal infections including *Candida*, *Aspergillus*, *Histoplasma*, and *Coccidioides*. Previously, we reported that **CWHM-974** has activity against *Candida albicans* isolates that are resistant to currently used antifungal drugs.^12^ We, therefore, submitted **CWHM-974** to The National Institutes of Health’s (NIH) antifungal susceptibility contract reference lab to explore its full spectrum of activity.

As shown in Table 1, the MICs for **CWHM-974** against pathogenic yeast were measured to be between 4 and 8 μg/mL (the exception was *C. albicans* SC5314). Consistent with our previously reported data,^12^**CWHM-974** was active against fluconazole-resistant *C. albicans*, *Candida auris*, and *Cryptococcus glabrata*. The MICs against filamentous molds, such as *Aspergillus fumigatus*, *Fusarium* spp., and *Rhizopus arrhizus*, were 2–4-fold higher than for pathogenic yeasts. A similar trend was also observed for the endemic human fungal pathogens *Histoplasma capsulatum*, *Blastomyces dermatitidis*, and *Coccidioides* spp. Notably, **CWHM-974** is active against *Coccidioides*, which causes nearly untreatable CNS infections in some patients. Taken together, this data indicates that PTZs have consistent antifungal activity toward a broad range of medically relevant fungi, including a number of organisms that are difficult or nearly impossible to treat with current drugs.

**Table 1:** Spectrum of Antifungal Activity for **CWHM-974**

species	number isolates tested	**CWHM-974** MIC (μg/mL)	posaconazole MIC (μg/mL)	voriconazole MIC (μg/mL)	fluconazole MIC (μg/mL)
*Candida parapsilosis*	4	4–8			4–16
*Candida krusei*	1	8			32
*Candida albicans*	3	8–16			>64
*Candida auris*	3	8			8 to >64
*Cryptococcus neoformans*	3	4			8–16
*Aspergillus fumigatus*	3	16		1 to >16	
*Paecilomyces variotii*	1	4	≤0.03	0.06	
*Apophysomyces* spp.	2	8–16	0.125–0.25		
*Saksenaea* spp.	2	1–4	0.06		
*Blastomyces dermatitidis*	3	16		0.125	
*Histoplasma capsulatum*	3	8		0.06	
*Coccidioides* spp.	3	4–8			32 to >64
*Rhizopus arrhizus*	3	16 to >64	0.125		
*Fusarium* spp.	3	16		2–16	
*Alternaria* spp.	1	16		4	
*Curvularia* spp.	1	16		0.5	
*Exserohilum* spp.	1	16		0.5	
*Scedosporium* spp.	3	8		0.5–1	

### **CWHM-974** Does Not Have Efficacy in Mouse Models of Disseminated Cryptococcosis or Candidiasis

As previously reported, **CWHM-974** has good CNS penetration and decreased toxicity in mouse models.^15^ Although a single dose of 10 mg/kg of **CWHM-974** leads to a brain maximum serum concentration (*C*_max_) just below the minimum inhibitory concentration (MIC) of *C. neoformans* (4 μg/mL),^15^ we hypothesized that multiple dosings could provide sufficiently high concentrations for efficacy. Therefore, CD-1 mice (10 mice/group) were infected with *C. neoformans* H99 by tail-vein infection to rapidly establish brain infection. The mice were treated with 10 mg/kg **CWHM-974** or vehicle by intraperitoneal (IP) injection for 4 days prior to sacrifice; IP injection is much less stressful for mice than oral gavage and also yields more consistent and reliable dosing. The mice were euthanized, and brain fungal burden was determined by quantitative plating. As shown in Figure S1A (Supporting Information), there was no difference in the fungal burden between the treatment and sham groups. Comparable results were observed at higher doses (50 mg/kg) in a model of disseminated candidiasis (Figure S1B). Based on these results, we employed additional medicinal chemistry-based optimization strategies on this general scaffold.

### Medicinal Chemistry Improvement Strategy

Our prior work led to the discovery of **CWHM-974**, which replaces the methylene linker between the PTZ core and the piperazine in fluphenazine with a bulky phenyl group (Figure 1).^12^**CWHM-974** is 2-fold more potent against *C. neoformans* and >8-fold more active against *Candida* spp.^16^ In addition, **CWHM-974** is significantly more selective against the dopamine and serotonin receptors. Selectivity against certain serotonin receptors, such as the 5HT_2C_ receptor, was improved, but only by ∼10-fold.

Docking studies of **CWHM-974** with the serotonin 5HT_2C_ receptor crystal structure (PDB 6BQH)^17^ (Figure S2), predicted that selectivity against the active site of the receptor may be improved upon by introducing steric bulk at the 7-position of the tricyclic PTZ ring. Furthermore, we hypothesized that modification of the PTZ ring with related tricyclic structures, such as carbazole or dibenzoazepine, may be tolerated and may influence selectivity against the 5HT_2C_ and related receptors. Thus, we began on the synthesis and antifungal SARs of replacement and substitution patterns of the PTZ ring of **CWHM-974** (**1**, Figure 1).

### Synthesis of PTZ Head Groups

**Scheme 1:**
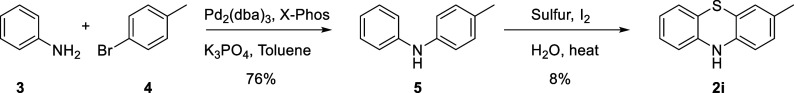
Synthesis of 3-Me PTZ Using Traditional PTZ Synthesis

PTZs **2a** and **2f**–**h** and related unsubstituted headgroups such as phenoxazine (POZ) **2b**, carbazole **2c**, diphenylamine **2d**, and dibenzoazepine **2e** were purchased to develop basic headgroup SAR (Figure S3). Since a limited number of PTZs are commercially available, and none are disubstituted variants, synthesis was required to obtain disubstituted head groups. Traditionally, PTZs were synthesized by using an iodine catalyst, where a substituted aniline and bromobenzene were reacted to afford a substituted diphenylamine.^18^ After isolation of this intermediate, the diphenylamine would be cyclized into the PTZ, using elemental sulfur and iodide at high temperatures.^18^ However, these reactions would result in only 10–50% yields, while the remaining 50–90% would be characterized as unreacted starting materials.^18^ Furthermore, the reaction would exhibit a lack of regioselective control, making development of SAR difficult.^19,20^ Thus, we limited our efforts with this approach to the mono 3-Me PTZ **2i** (Scheme 1). Toward this end, aniline **3** was reacted with bromoarene **4** using Buchwald–Hartwig reaction conditions to get diarylamine intermediate **5** in good yield. Then **5** was reacted with sulfur and iodide to produce the 3-Me PTZ **2i** in low yield.

**Scheme 2:**
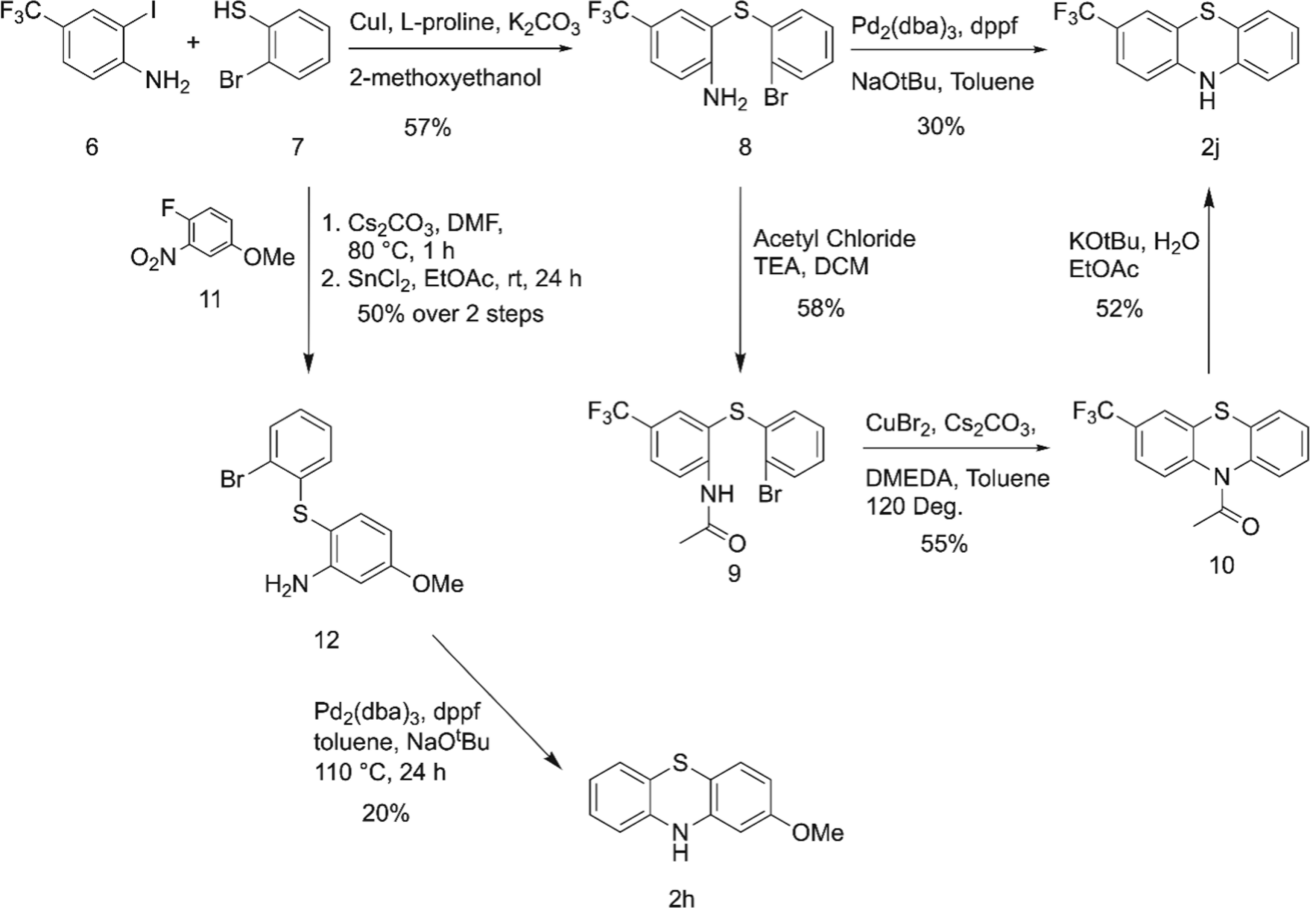
Synthesis of 4-CF_3_ and 3-OMe PTZs

Several research groups have reported approaches to condense aryl halides, arylthiols, and aryl anilines in various combinations to construct PTZs via a coupling reaction, followed by cyclization.^21−26^ We investigated a number of these approaches but consistently found the chemistry to stall prior to the second cyclization step, in contrast to the published methods. One example is shown in Scheme 2, using CuI and l-proline to catalyze the reaction of 2-iodoanilines and 2-bromobenzenethiols to synthesize PTZs. Here, iodoaniline **6** was reacted with bromothiophenol **7** using Ullmann reaction conditions to afford intermediate **8**.^19,23^ We expected the two-step conversion to **2j** to occur during this single step. However, although published methods were followed, the conversion of **8** into PTZ **2j** was unsuccessful and led to an incomplete synthesis. Only about a third of the theoretical amount of PTZ **2j** was isolated, and the remaining substance was uncyclized **8**. Thus, to overcome this issue, Buchwald conditions were employed to convert **8** into **2j**.^19^

Due to incomplete cyclization of the tricyclic PTZ structure from diarylthioether precursors, we developed a stepwise method (Scheme 2) to synthesize PTZ derivatives that had been difficult to synthesize using the previously stated methods. Diphenyl thioether **8** was acylated to furnish amide **9**.^27^ Following acylation, another Ullmann-type coupling reaction on **9**,^28^ using Cu(II)Br, displaced the bromine and formed the tricyclic PTZ ring structure seen on **10**. Deacetylation was completed by using potassium *tert*-butoxide, followed by an aqueous workup, giving the desired PTZ **2j**.^29^ Using an alternative pathway toward PTZ **2h**, intermediate **12** was synthesized via a Cs_2_CO_3_-promoted aromatic nucleophilic substitution reaction (Scheme 2) between **7** and **11**, followed by reduction of the nitro group using SnCl_2_ with a satisfactory yield of 50% over two steps. **12** was converted to **2h** using the Pd-catalyzed intramolecular Buchwald reaction in 20% yield.

**Scheme 3:**
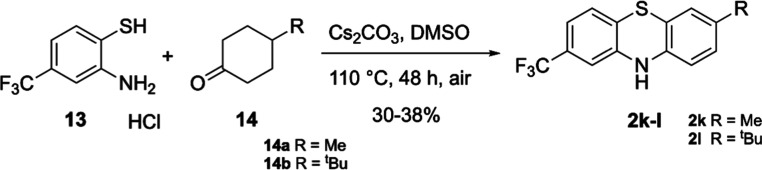
Synthesis of 2,7-Disubstituted PTZs

As illustrated in Figure S2, we predicted that large R groups in the 7-position would result in improved selectivity. To evaluate this hypothesis, we prepared target 2,7-disubstituted PTZs **2k**–**l** with 7-Me and 7-*t*-butyl groups by applying an alternative approach described by Liao and co-workers^30^ since starting materials for the approaches described above were not readily available (Scheme 3). Condensation of thioaniline **13** with cyclohexanones **14a**–**b** proceeded as described in dimethyl sulfoxide (DMSO) with cesium carbonate to give the novel disubstituted PTZs **2k**–**l** in modest yields.

**Scheme 4:**
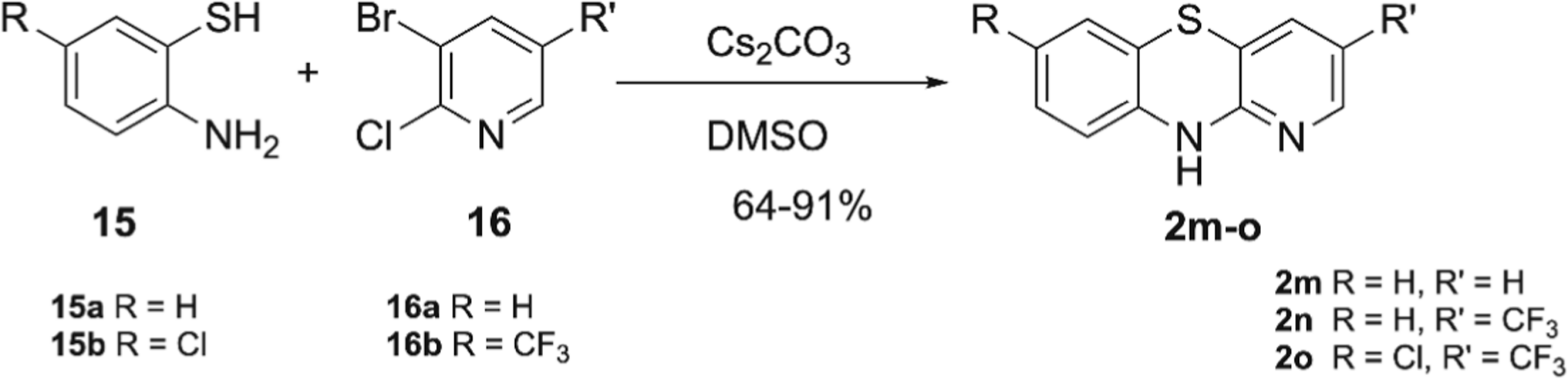
Synthesis of Aza-PTZs

All previously synthesized PTZs consisted of two fused phenyl rings. However, the addition of one or more polar atoms in the aromatic ring can lead to a change in potency and biological activity. To test and further explore the effects of incorporating heterocycles in PTZs, the following method was employed (Scheme 4). Here, thioanilines **15a**–**b** were reacted with 3-bromo-2-chloropyridines **16a**–**b** using a simple displacement reaction to produce aza-PTZs **2m**–**o**. Unlike the previous cyclization reactions, these were completed with ease and high yields in a single step. The regiochemistry of **2m**–**o** was assigned and confirmed via nuclear Overhauser effect spectroscopy (NOESY) and proton nuclear magnetic resonance (^1^H NMR).

### Synthesis of Final Analogs

**Scheme 5:**
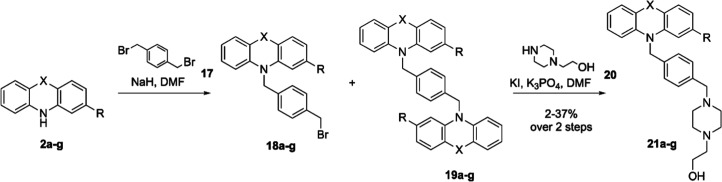
Initial Route to Final Analogs **21a–g**

Following the synthesis of the PTZ headgroups, the focus was directed toward adding the 1,4-xylene linker and piperazine ethanol moiety. The first method used to prepare **21a**–**g** introduced the linker and tail in a two-step synthesis. This procedure was previously described for the synthesis of **CWHM-974**(12) (Scheme 5). The commercially available anilines and PTZs **2a**–**g** were reacted with dibromo-*p*-xylene after the treatment with a base. However, this synthetic route led to the formation of dialkylated byproducts **19a**–**g** which were difficult to control and separate from the desired bromides **18a**–**g**, despite variations in reaction temperature, number of equivalents, rate of substrate addition, and order of reagent addition. Only after the addition of the piperazine ethanol **20** was purification of the desired products **21a**–**g** from the dialkylated byproducts **19a**–**g** successful.

**Scheme 6:**
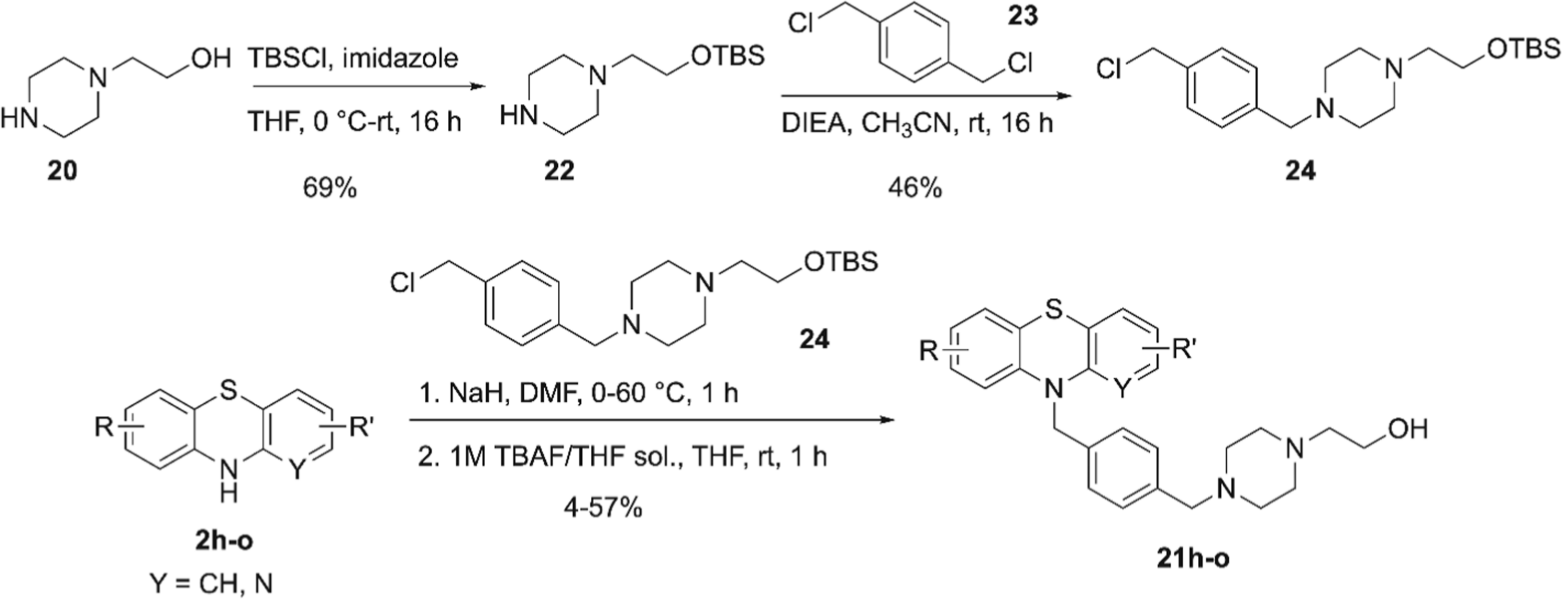
Synthesis of Intermediate **24** and an Improved Route to Final Analogs

Given the challenges with the original alkylation route, a new route was developed (Scheme 6). We started with selective protection of piperazinyl ethanol **20** to afford *tert*-butyldiphenylsilyl (TBS) ether **22**(31) followed by the addition of **22** to the 1,4-bis(chloromethyl)benzene **23** under basic conditions to furnish the protected benzyl chloride **24**. PTZs **2h**–**o** were then deprotonated with sodium hydride and reacted with benzyl chloride intermediate **23**. Deprotection of the TBS group using tetra-*n*-butylammonium fluoride (TBAF) solution^32^ resulted in the formation and isolation of the desired analogs **21h**–**o**.

### Antifungal PTZ SAR

The antifungal activity of the PTZ analogs was determined using standard Clinical and Laboratory Standards Institute (CLSI) microbroth dilution assays and reference strains for *C. neoformans* (H99) and *C. albicans* (SC5314),1 as previously described.^33^ Fluconazole was used as a reference control. Results are presented in Table 2.

**Table 2:**
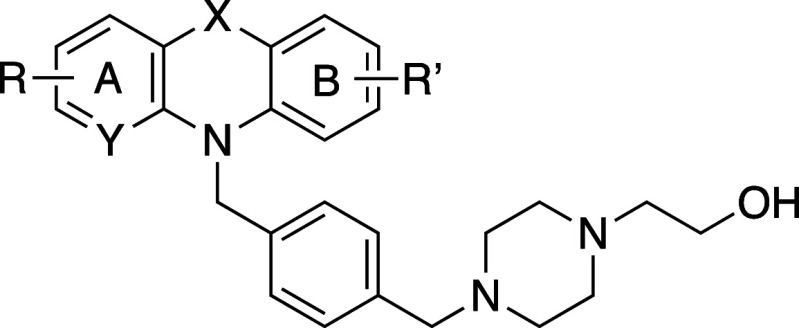
Antifungal SAR for Novel PTZ Analogs

compound	SLU	X	Y	A-ring R	B-ring R′	*Cn* H99 MIC (μg/mL)^a^	*Ca* SC5314 MIC (μg/mL)^a^	*c* Log *P*^b^
**fluconazole**	N/A	N/A	N/A	N/A	N/A	2	≤0.125	0.6
**fluphenazine**	N/A	S	CH	2-CF_3_	H	8	>50	4.9
**CWHM-974**	N/A	S	CH	2-CF_3_	H	4	8	6.1
Unsubstituted PTZ Headgroup Comparison								
**21a**	10026	S	CH	H	H	4	16	5.0
**21b**	10027	O	CH	H	H	8	16	4.1
**21c**	10028	bond	CH	H	H	8	32	3.0
**21d**	10030	H,H	CH	H	H	8	32	4.1
**21e**	10031	CH_2_CH_2_	CH	H	H	4–8	32	4.5
Monosubstituted PTZs								
**21f**	10029	S	CH	2-Cl	H	4	8	5.6
**21g**	10812	S	CH	2-CN	H	4	32	4.7
**21h**	11018	S	CH	2-OMe	H	4	8–16	5.1
**21i**	11017	S	CH	3-Me	H	2	8	5.5
**21j**	11109	S	CH	3-CF_3_	H	2	4	6.0
Disubstituted PTZs								
**21k**	11079	S	CH	2-CF_3_	7-Me	2	2–4	6.5
**21l**	11044	S	CH	2-CF_3_	7-*t*Bu	1	>128	7.6
Aza PTZs								
**21m**	11081	S	N	H	H	8	32	4.7
**21n**	11110	S	N	3-CF_3_	H	2	4	5.7
**21o**	11156	S	N	3-CF_3_	7-Cl	2	4	6.3

a The MIC values were determined at 24 h incubation for *C. albicans* and 72 h incubation for *C. neoformans*.

b *c* Log *P* represents an estimate of lipophilicity and is the log of the partition coefficient as calculated in CDD Vault using the ChemAxon fragment approach (www.collaborativedrug.com).

Initial SAR for the PTZ headgroup was developed from **21a**–**e** wherein the importance of the sulfur atom (X) was investigated. Unsubstituted PTZ comparator **21a** was nearly equipotent to **CWHM-974**, allowing for the direct comparison of analogs varying in X. POZ **21b** was about 2-fold less potent than PTZ **21a**. Other variations to the PTZ headgroup (**21c**–**e**) were tolerated for *C. neoformans* but resulted in a bit less potency against *C. albicans*. Since the desire was to develop a broad-spectrum antifungal, we settled on PTZ as the optimal headgroup.

Generally, unsubstituted PTZs (**21a**–**e**, **21m**) exhibited a lower level of potency when compared with their substituted counterparts (**CWHM-974** and **21f**–**l**,**n**,**o**) in the same antifungal assays. Monosubstituted analogs (**21f**–**j**) exhibited a similar potency in *C. neoformans* to that of **CWHM-974**. However, these analogs decreased 2–4-fold in potency against *C. albicans*. More specifically, substitutions at the 2-position (**21f**–**h**) led to comparable results as those seen in **CWHM-974** against *C. neoformans*, while substitutions at the 3-position (**21i**–**j**) led to a 2-fold increase in potency against *C. neoformans*, regardless of the electronic character of the substituent. This indicated that a substitution off the 3-position was preferred for potency.

Disubstituted analogs **21k**–**l** showed an overall improvement in potency against *C. neoformans*. Both 7-Me **21k** and 7-*t*Bu **21l** exhibited a 2–4-fold potency increase when compared to **CWHM-974**. Two different electron-donating substituents were installed in the 7-position, while the 2-position was kept consistent with an electron-withdrawing trifluoromethyl group. Surprisingly, analysis of these compounds in *C. albicans* led to two significantly opposite results: 7-Me **21k** was 2-fold more potent, while 7-*t*Bu **21l** had a steep decrease in potency against the same fungus. This latter result suggests that there is some aspect of specific binding activity that is not tolerated by a large *t*-butyl group.

Furthermore, a pyridine substitution in the PTZ scaffold, **21m**–**o**, not only modestly improved potency relative to **CWHM-974**, but it also led to a significantly more convenient synthetic process in the lab. As discussed previously, the unsubstituted aza-PTZ, **21m**, was 2-fold less potent against *C. neoformans* and 4-fold less potent against *C. albicans*. However, the addition of one or two substituents (**21n** and **21o**) restored potency. Here, both electron-withdrawing groups in the 3-position and the addition of the chloro-substituent in the 7-position led to an increase in potency by 2-fold in both *C. neoformans* and *C. albicans*. These discoveries make mono- and di-substituted aza-PTZ analogs particularly attractive for future optimization.

### Selectivity Profiling of 7-Alkyl PTZ Analogs

The scaffold (**CWHM-974**) was previously evaluated by in vitro cytotoxicity data and neuroreceptor activity, which is the main source of dose limiting toxicity in the animals.^12,15^ Therefore, we focused on neuroreceptor activity for this study. To assess our selectivity hypothesis, disubstituted analogs **21k** (7-Me analog) and **21l** (7-*t*Bu analog) were assayed against an extensive panel of dopamine and serotonin receptors in radioligand binding assays at the National Institute of Mental Health’s Psychoactive Drug Screening Program (PDSP). Inhibitory concentration (IC_50_) values were determined for compounds with >50% binding affinity at 10 μM (Table 3). Previously, we showed that **CWHM-974** is significantly more selective against serotonin and dopamine receptors than trifluoperazine, due in part to the presence of the phenyl linker.^12^ Unfortunately, disubstituted analogs **21k**–**l** did not support our 5-HT_2c_ selectivity hypothesis, with **21l** maintaining and **21k** only modestly (3-fold) improving selectivity against this receptor. In fact, we saw somewhat of an erosion in selectivity with **21k**–**l**, relative to **CWHM-974**, particularly for receptors 5-HT_1B_, 5-HT_1D_, D1, and D3.

**Table 3:** Receptor Binding Selectivity Data

receptor	radioligand binding assay IC_50_ (nM)^a^			
	trifluoperazine^b^	**CWHM-974**^b^	**21k**	**21l**
5-HT_1A_	392	10,000	10,000	1444
5-HT_1B_	1210	10,000	774	473
5-HT_1D_	1266	10,000	683	385
5-HT_1E_	10,000	10,000	10,000	1507
5-HT_2A_	11	325	1027	897
5-HT_2B_	112	347	112	213
5-HT_2C_	63	464	1450	556
5-HT_3_	581	10,000	10,000	10,000
5-HT_5A_	1795	10,000	10,000	3637
5-HT_6_	235	2470	10,000	10,000
5-HT_7A_	10,000	10,000	3873	1708
D1	44	1403	654	75
D2	541	10,000	10,000	728
D3	29	2307	535	386
D4	416	10,000	10,000	10,000
D5	70	1336	1601	1769
HERG	n.d.	n.d.	1041	1651

a Compounds with <50% inhibition at 10 μM are reported as >10,000 nM.

b Data from ref (12).

## Conclusions

With only three classes of antifungal drugs and the rise of drug-resistant fungi, development of novel antifungal drugs represents a significant unmet medical need. Toward this end, we have shown that the antifungal activity of PTZ-based **CWHM-974** extends beyond previously reported activity against *C. neoformans* and *C. albicans* to include fluconazole-resistant *C. albicans*, *C. auris*, and *C. glabrata*, filamentous molds such as *A. fumigatus*, *Fusarium* spp., and *R. arrhizus*, endemic human fungal pathogens *H. capsulatum*, *B. dermatitidis*, and *Coccidioides* spp. Thus, PTZs have consistent antifungal activity toward a broad range of medically relevant fungi, including organisms that are difficult or nearly impossible to treat with available drugs.

**CWHM-974** is CNS-penetrant, reaching *C*_max_ concentrations in the mouse brain, which approach its MIC value of 4 μg/mL against *C. neoformans* when dosed at 10 mg/kg. Unfortunately, **CWHM-974** did not exhibit in vivo efficacy in either *C. neoformans* or *C. albicans* mouse infection models at 10 or 50 mg/kg, respectively, leading us to conclude that more potent analogs need to be identified to establish in vivo efficacy.

For this reason, we embarked on an effort to develop SAR on the PTZ ring to improve antifungal potency and selectivity. PTZ head groups were synthesized by various approaches, permitting synthesis and determination of MIC values against *C. neoformans* and *C. albicans* for 15 novel PTZ analogs. Replacement of the PTZ headgroup with related tri- and bi-cyclic head groups was tolerated, but these replacements did not result in improved antifungal activity. Six PTZ analogs with mono 3-substitution, 2,7- and 3,7-disubstitution patterns were identified as being 2- to 4-fold more potent against these organisms. Aza-PTZs were also tolerated and, with appropriate substitution, resulted in potent antifungals with a moderate reduction in lipophilicity and more facile chemical synthesis. This study focused on the phenothiazine headgroup but the linker and basic amine tail remain unoptimized for PTZs and the other tri- and bicyclic head groups identified, suggesting the potential for further improvements in potency, selectivity and efficacy with additional SAR.

The recent publication of the 5HT_2c_ X-ray crystal structure provided a hypothesis regarding enhancement of selectivity versus 5HT_2c_ by incorporation of a substituent in the 7-position. This hypothesis was evaluated with two analogs (**21k** and **21l**). One analog did display modest selectivity improvement versus **CWHM**-**974**, but its overall selectivity profile versus a panel of serotonin and dopamine receptors was not improved. Further enhancement of selectivity remains a significant challenge and will likely require more extensive modification to the scaffold than our hypothesis predicted.

Overall, the broad-spectrum antifungal activity and reduced neuroreceptor affinity of PTZ-based **CWHM-974** and its analogs encourage continued optimization of this promising scaffold to develop a novel antifungal therapeutic drug. In particular, more potent analogs are needed in order to improve the ratio between antifungal potency and neuroreceptor affinity as well as in vivo efficacy. Further work to improve antifungal potency and in vivo efficacy in this series will be reported accordingly.

## Materials and Methods

### Compounds

The synthesis of **CWHM-974** has been reported previously.^12^ The synthesis of novel compounds is reported in the Supporting Information section. All final compounds are ≥95% pure by high-pressure liquid chromatography (HPLC) and have their identities confirmed by high resolution mass spectrometry (HRMS) and proton NMR (^1^H NMR).

### Statement of Animal Ethics

The animal experiments described in this work were approved by the Institutional Animal Care and Use Committee at the University of Iowa. For all studies, mice were housed 5 per cage and had access to food and water ad libitum.

### Antifungal Susceptibility Testing

MIC values were determined using the CLSI guidelines, as previously described.^33^*C. albicans* (SC5314) and *C. neoformans* (H99) were cultured overnight in 3 mL yeast peptone 2% dextrose (YPD) at 30 °C, then washed twice in sterile phosphate-buffered saline (PBS). 2-fold serial dilutions of each drug were prepared in RPMI + MOPS pH 7 (Gibco RPMI 1640 with l-glutamine [11875-093] and 0.165 M MOPS); then 1 × 10^3^ cells were added per well. Plates were incubated at 37 °C for 24 h (*C. albicans*) or 72 h (*C. neoformans*). The susceptibilities of the species described in Table 2 were performed under CLSI conditions at the University of Texas Health Sciences Center at San Antonio under NIH contract number: 75N93019D00022.

### In Vivo Efficacy Studies

For in vivo studies, **CWHM-974** was formulated with 5% DMSO in 20% Kolliphor RH-40 (Sigma, 07076)/D5W (5% dextrose in water).

#### Disseminated Candidiasis Model

*C. albicans* SN425 was cultured in 25 mL YPD for 24 h. Then 25 μL were subcultured into fresh YPD (25 mL) for an additional 24 h. This was repeated for a total of two passages over 72 h. Yeast cells were washed with sterile PBS and then enumerated and diluted to 1.9 × 10^6^ colony-forming units per milliliter (CFU/mL) in PBS. Twenty CD-1 females (25–30 g, Envigo) were inoculated with 200 μL (5 × 10^6^ CFU/mL) of SC5314 via lateral tail vein (LTV). Starting at 1 h post inoculation (hpi), mice were dosed with 50 mg/kg **CWHM-974** or an equivalent volume of vehicle (*n* = 10 mice/group) every 24 h for 72 h. Kidneys were harvested 72 hpi and homogenized in 1 mL sterile PBS. Then 10-fold dilutions of organ homogenate were prepared in PBS and plated on YPD. Each organ was plated in technical duplicate, followed by incubation at 30 °C for 24 h, for colony enumeration. Results are presented in Figure S1.

#### Disseminated Cryptococcosis Model

*C. neoformans* H99 was cultured in 25 mL YPD for 48 h. Then the cells were washed three times with sterile PBS, enumerated with a hemocytometer, and diluted to 1.9 × 10^6^ CFU/mL in sterile PBS. Twenty CD-1 females (25–30 g, Envigo) were inoculated with 3.8 × 10^5^ CFUs in 200 μL via LTV. Starting after 1 hpi, mice were dosed with 10 mg/kg **CWHM-974** or vehicle every 12 h for 72 h via IP injection (*n* = 10 per group). Brains were harvested 72 hpi, homogenized in 1 mL sterile PBS, then 10-fold dilutions were prepared in sterile PBS. Each dilution was plated on YPD in technical duplicate, and colonies were enumerated after 48 h incubation at 30 °C. Results are presented in Figure S1.

## Supporting Information Available

Figures S1–S38, animal study results, docking study, experimental procedures for the synthesis and characterization of compounds (PDF)

## Abbreviations

aza-PTZ: azaphenothiazine
*C*_max_: maximum serum concentration
CFU: colony-forming unit
CFU/mL: colony-forming units per milliliter
*c* Log *P*: calculated Log *P*
CLSI: Clinical and Laboratory Standards Institute
CNS: central nervous system
D: class of dopamine receptors
DMSO: dimethyl sulfoxide
D5W: 5% dextrose in water
HIV/AIDS: human immunodeficiency virus/acquired immunodeficiency syndrome
HPLC: high-pressure liquid chromatography
hpi: hour(s) post inoculation
HRMS: high resolution mass spectrometry
IC_50_: half maximal inhibitory concentration
IP: intraperitoneal
LTV: lateral tail vein
MIC: minimum inhibitory concentration
NIH: The National Institutes of Health
NIMH: National Institute of Mental Health
NMR: nuclear magnetic resonance
NOESY: nuclear Overhauser effect spectroscopy
PBS: phosphate buffered saline
PDSP: Psychoactive Drug Screening Program
POZ: phenoxazine
PTZ: phenothiazine
SAR: structure–activity relationship
spp.: several species
TBAF: tetra-*n*-butylammonium fluoride
TBS: *tert*-butyldiphenylsilyl
WHO: The World Health Organization
YPD: yeast peptone 2% dextrose
^1^H NMR: proton nuclear magnetic resonance
5HT: class of serotonin receptors
