# Supplementary material for: Antifungal Structure–Activity Relationship Studies of Broad-Spectrum Phenothiazines
Source: ACS Omega. 2025 Apr 29;10(18):18347–55. doi: 10.1021/acsomega.4c09833 (PMC12079586; doi:10.1021/acsomega.4c09833)
Supplement: Supplementary file 1 — ao4c09833_si_001.pdf [file ao4c09833_si_001.pdf]

Supporting Information

Antifungal structure-activity relationship studies of broad-spectrum phenothiazines

Samantha C. Brosend,[a] Soumitra Guin,[a] Gregory Giovine,[a] Carlos Gadalla,[a] Miguel A. Campos,[a] Alisa Mara,[a] Nicholas G. Jentsch,[a] Haresh Thakellapalli,[a] Kathryn M. Alden,[b,c] Sarah R. Beattie,[b,c] Damian J. Krysan,[b,c]* Marvin J. Meyers[a,d]*

[a] Department of Chemistry, School of Science and Engineering, Saint Louis University, Saint Louis, MO 63103
[b] Department of Pediatrics, Carver College of Medicine, University of Iowa, Iowa City, IA 52242
[c] Department of Molecular Physiology and Biophysics, University of Iowa, Iowa City, IA 52242
[d] Institute for Drug and Biotherapeutic Innovation, Saint Louis University, St. Louis, MO 63103

Corresponding Authors
* E-mail: MJM: marvin.j.meyers@slu.edu. 3501 Laclede Ave, Monsanto Hall, Saint Louis, MO 63103.
DJK: damian-krysan@uiowa.edu. Med Labs 2020, University of Iowa, 200 S. Grand Ave, Iowa City, IA 52242.

Table of Contents

Fig. S1. Mouse models of disseminated cryptococcosis or candidiasis S2

Fig. S2. Docking study of CWHM-974 in the serotonin $5HT_{2C}$ receptor S3

Figure S3. Commercially available PTZ head groups S3

Experimental Procedure for the Synthesis of Compounds S4

Fig. S4-S38. Supplementary Analysis and Compound Spectra S18

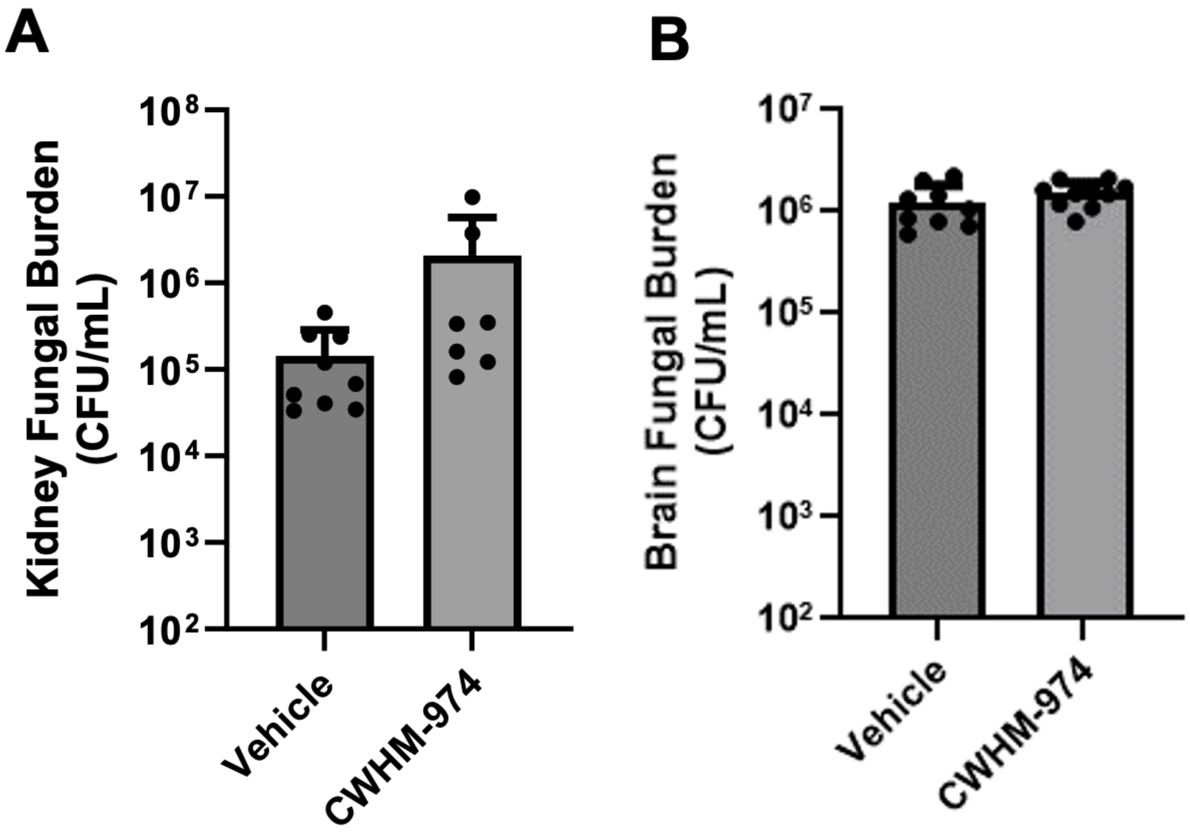

Figure S1. CHWM-974 is not efficacious in mouse models of disseminated cryptococcosis or candidiasis. A. CD-1 mice (n=10/group) were inoculated with *C. albicans* strain SN425 by tail-vein injection and treated daily with 50 mg/kg **CHWM-974** or vehicle. Brain fungal burden was determined by quantitative plating 72 hours post-infection. NS indicates no statistical difference by Mann-Whitney test. **B**. CD-1 mice (n=10/group) were inoculated with *C. neoformans* strain H99 by tail-vein injection and treated daily with 10 mg/kg **CHWM-974** or vehicle. Brain fungal burden was determined by quantitative plating 72 hours post-infection. NS indicates no statistical difference by Mann-Whitney test.

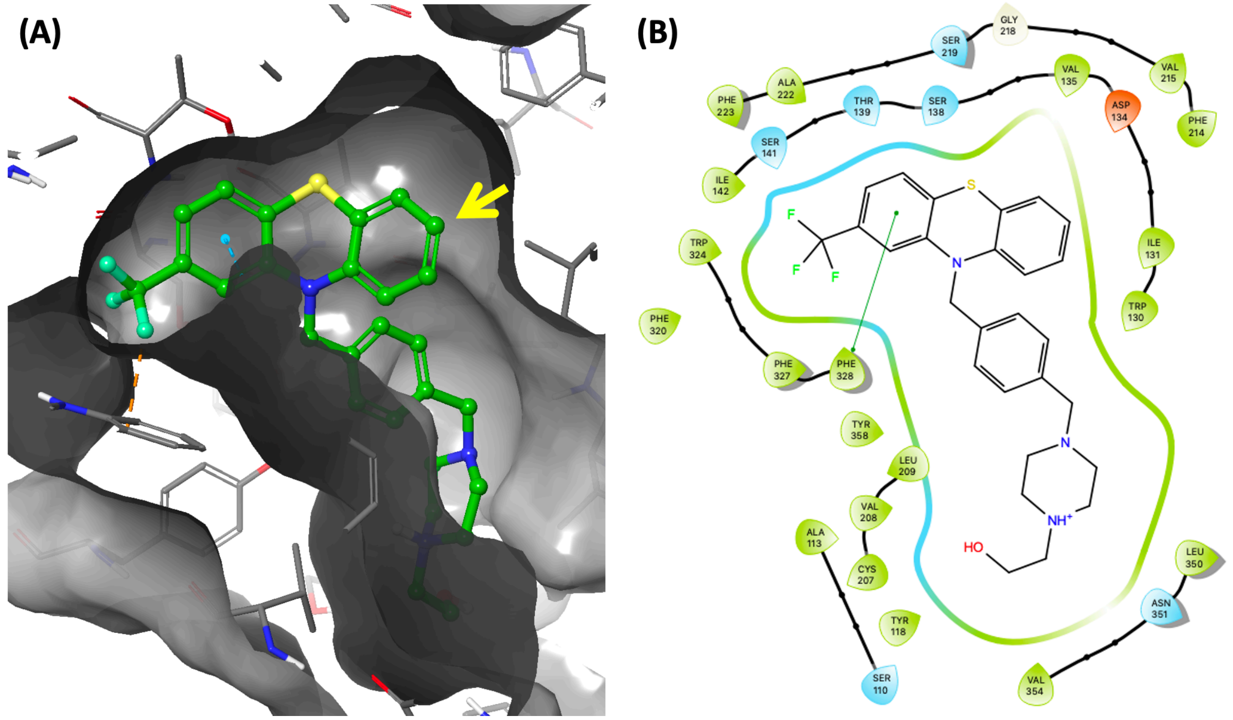

Figure S2. Docking study of CWHM-974 in the serotonin 5HT$_{2C}$ receptor. CWHM-974 was docked into the 5HT$_{2C}$ receptor (PDB 6BQH). (A) The yellow arrow illustrates the predicted trajectory for substitution to reduce affinity for the 5HT$_{2C}$ receptor due to potential to clash with the protein. (B) Ligand interaction diagram.

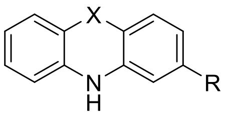

2

2a X = S, R = H
2b X = O, R = H
2c X = bond, R = H
2d X = H/H, R = H
2e X = CH_2CH_2, R = H
2f X = S, R = Cl
2g X = S, R = CN
2h X = S, R = OMe

Figure S3. Commercially available PTZ head groups.

Experimental Procedure for the Synthesis of Compounds

General: Commercially available reagents and solvents were used without further purification unless stated otherwise. HPLC and LC-MS analyses were performed on an Agilent 1260 HPLC/MSD electrospray mass spectrometer in positive and negative ion mode with a scan range of 100-1000 Da. Preparative normal-phase chromatography was performed on a CombiFlash Rf+ (Teledyne Isco) with SiliaFlash F_{60} 40-63 μm (230-400 mesh) silica gel (SiliCycle Inc.). Preparative reverse-phase HPLC was performed on a CombiFlash Rf+ (Teledyne Isco) equipped with RediSep Rf Gold pre-packed C18 cartridges and on an ACCQPrep HP150 (Teledyne Isco) equipped with 10 x 250mm, 20 x 250mm, and 30 x 250mm C18 RediSep Prep HPLC columns and an acetonitrile/water/0.1% formic acid modifier gradient. NMR spectra were recorded on Bruker 400, and 700 MHz spectrometers. The signal of the deuterated solvent was used as the internal reference. Chemical shifts (δ) are given in ppm and are referenced to a residual not fully deuterated solvent signal. Coupling constants (J) are given in Hertz (Hz). HRMS spectra were recorded on an ABSciex 5600+ instrument. All final compounds for biological testing were purified to ≥ 95% as determined by LCMS or HPLC-UV absorbance unless noted otherwise.

4-Methyl-N-phenylaniline (5): In a heat-dried reaction vial, $Pd_2(dba)_3$ (220 mg, 0.24 mmol), X-

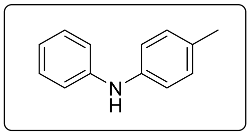

Phos (400 mg, 0.86 mmol), and potassium phosphate (3.2 g, 15 mmol) were dissolved in toluene (10 mL) before backfilling with inert gas. To the mixture, **3** (0.97 mL, 11 mmol) and **4** (1.8 g, 11 mmol) were added. The mixture was then heated to 110 °C and stirred for 20 hours. After completion, the crude mixture was cooled to room temperature and diluted with EtOAc before being washed with water and brine. The organic layer was then dried over anhydrous $MgSO_4$ and concentrated under vacuum to afford the crude material, a yellow oil. This was then purified by normal phase silica gel chromatography, and fractions of interest were combined before drying under vacuum to afford the title compound as a dark yellow solid, (1.8 g, yield 76%, purity 81%); **^{1}H NMR** (400 MHz, $CDCl_3$) δ 7.24 (d, J = 7.83 Hz, 2H),, 7.08 - 7.12 (m, 2H), 7.03 (d, J = 2.45 Hz, 2H), 7.01 (d, J = 2.45 Hz, 2H), 6.89 (t, J = 7.34 Hz, 1H), 5.61 (br. s., 1H), 2.32 (s, 3H). **LCMS** $[M+H]^+$ 184.

3-Methyl-10H-phenothiazine (2i): In a microwave vial, iodine (140 mg, 0.55 mmol), sulfur (350 mg, 11 mmol), and **5** (1.0 g, 5.5 mmol) were dissolved in deionized water (3 mL). The resulting mixture was then heated at 190 °C under microwave irradiation for 20 min. Due to starting material still being present, the microwave vial was loaded with an additional amount of iodine (0.1 eq) and heated for an additional 2 hours at 190 °C. After completion, the crude reaction mixture was cooled to room temperature and dissolved in chloroform and separated from the water phase. The organic layer was concentrated under vacuum and purified using reverse phase column chromatography using RediSep Rf Gold pre-packed C18 cartridges with a 5 to 100% acetonitrile/water/0.1% formic acid gradient. Fractions were then lyophilized to afford the title compound, (94 mg, yield 8%, purity >99%); ^{1}**H NMR** (400 MHz, Methanol-d_4) δ 6.93 (dt, J = 1.47, 7.70 Hz, 1H), 6.86 (dd, J = 1.34, 7.70 Hz, 1H), 6.72 - 6.78 (m, 1H), 6.69 - 6.72 (m, 2H), 6.61 (dd, J = 1.10, 7.95 Hz, 1H), 6.53 (d, J = 7.82 Hz, 1H), 2.16 (s, 3H). **LCMS** $[M+H]^+$ 214.

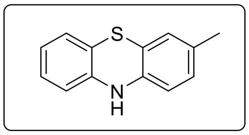

2-((2-Bromophenyl)thio)-4-(trifluoromethyl)aniline (8): In a heat-dried reaction vial, **6** (1.0 g, 3.5 mmol), copper iodide (98 mg, 0.70 mmol), L-proline (160 mg, 1.4 mmol), and potassium carbonate (2.4 g, 17 mmol) were dissolved in 2-methoxyethanol (15 mL) before backfilling with inert gas. To the mixture, **7** (0.46 mL, 3.8 mmol) was then added. The mixture was heated to 90 °C and stirred for 16 hours. After completion, the crude mixture was cooled to room temperature and diluted with EtOAc before being washed with water and brine. The organic layer was then dried over anhydrous $MgSO_4$ and concentrated under vacuum to afford the crude material, a peach-orange oil. This was then purified by normal phase silica gel chromatography, and fractions of interest were combined before drying under vacuum to afford the title compound as a yellow oil, (821 mg, yield 57%, purity 84%); ^{1}**H NMR** (400 MHz, DMSO-d_6) δ 7.63 (dd, J = 1.35, 7.95 Hz, 1H), 7.60 (d, J = 1.47 Hz, 1H), 7.53 (dd, J = 2.20, 8.80 Hz, 1H), 7.23 - 7.28 (m, 1H), 7.09 (dt, J = 1.47, 7.70 Hz, 1H), 6.97 (d, J = 8.56 Hz, 1H), 6.54 (dd, J = 1.47, 8.07 Hz, 1H), 6.17 (s, 2H). **LCMS** $[M+H]^+$ 347.

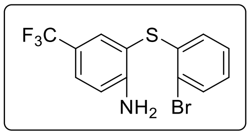

3-(Trifluoromethyl)-10H-phenothiazine (2j): In a heat-dried reaction vial, **8** (500 mg, 1.4 mmol), $Pd_2(dba)_3$ (400 mg, 0.43 mmol), dppf (400 mg, 0.72 mmol), and sodium *tert*-butoxide (420 mg, 4.3 mmol) were dissolved in toluene (10 mL) before backfilling with inert gas. The mixture was heated to 120 °C and

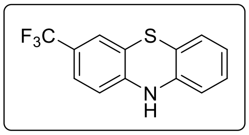

stirred for 16 hours. After completion, the crude mixture was cooled to room temperature and diluted with EtOAc before being washed with water and brine. The organic layer was then dried over anhydrous $MgSO_4$ and concentrated under vacuum to afford the crude material, a brown oil. This was then purified by normal phase silica gel chromatography, and fractions of interest were combined before drying under vacuum to afford the title compound as a red-orange solid, (132 mg, yield 30%, purity 89%); **^{1}H NMR** (400 MHz, DMSO-d_6) δ 9.02 (s, 1H), 7.45 - 7.49 (m, 1H), 7.29 (d, *J* = 8.31 Hz, 1H), 7.22 (s, 1H), 6.99 - 7.04 (m, 1H), 6.92 (d, *J* = 6.85 Hz, 1H), 6.78 (dd, *J* = 7.95, 19.20 Hz, 1H), 6.69 (d, *J* = 8.07 Hz, 1H). **LCMS** $[M-H]^-$ 266.

N-(2-((2-bromophenyl)thio)-4-(trifluoromethyl)phenyl)acetamide (9): In a reaction vial, **8**

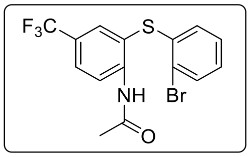

(280 mg, 0.80 mmol) and anhydrous Et_3N (0.17 mL, 1.2 mmol) were dissolved in anhydrous DCM (5 mL). The reaction was cooled to 0 °C and stirred for 15 minutes. Then, acetyl chloride (0.069 mL, 0.97 mmol) was added dropwise to the mixture, turning it a bright yellow color. Once addition was complete, the vessel and contents were warmed to room temperature and stirred for 12 hours. Upon reaction completion, the mixture was diluted and washed with DCM (3x) before being dried over anhydrous $MgSO_4$ and concentrated under vacuum to afford the crude material, a chunky yellow solid. This was then purified by normal phase silica gel chromatography, and fractions of interest were combined before drying under vacuum to afford the title compound as a yellow crystalline solid, (283.1 mg, yield 58%, purity 64%); **^{1}H NMR** (400 MHz, DMSO-d_6) δ 9.75 (s, 1H), 8.00 (d, *J* = 8.50 Hz, 1H), 7.78 (dd, *J* = 2.00, 8.63 Hz, 1H), 7.72 (dd, *J* = 1.38, 7.88 Hz, 1H), 7.58 (d, *J* = 1.88 Hz, 1H), 7.30 - 7.35 (m, 1H), 7.23 (dt, *J* = 1.56, 7.66 Hz, 1H), 6.90 (dd, *J* = 1.56, 7.82 Hz, 1H), 2.05 (s, 3H). **LCMS** $[M+H]^+$ 389.

1-(3-(Trifluoromethyl)-10H-phenothiazin-10-yl)ethan-1-one (10): In a heat-dried reaction vial,

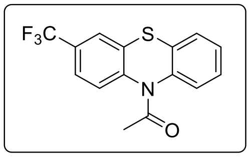

9 (200 mg, 0.51 mmol), copper bromide (3.7 mg, 0.026 mmol), cesium carbonate (420 mg, 1.3 mmol), and DMEDA (0.11 mL, 1.0 mmol) were dissolved in toluene (4 mL) before backfilling with inert gas. The mixture was heated to 120 °C and stirred for 2 hours. After completion, the crude mixture was cooled to room temperature and filtered through a Celite plug. The filtrate was then extracted using EtOAc before being washed with water and brine. The organic layer was then dried over anhydrous $MgSO_4$ and concentrated under vacuum to afford the crude material, a yellow oil.

This was then purified by normal phase silica gel chromatography, and fractions of interest were combined before drying under vacuum to afford the title compound as a transparent yellow oil, (88 mg, yield 55%, purity >99%); **^{1}H NMR** (400 MHz, DMSO-d_6) δ 7.98 (d, J = 1.38 Hz, 1H), 7.81 - 7.86 (m, 1H), 7.73 - 7.77 (m, 1H), 7.67 (dd, J = 1.00, 8.00 Hz, 1H), 7.59 (dd, J = 1.38, 7.75 Hz, 1H), 7.44 (dt, J = 1.50, 7.69 Hz, 1H), 7.32 - 7.37 (m, 1H), 2.16 (s, 3H). **LCMS** $[M+H]^+$ 310.

3-(Trifluoromethyl)-10H-phenothiazine (2j): In a heat-dried reaction vial, **10** (85 mg, 0.27 mmol) was dissolved in anhydrous EtOAc (1.5 mL) before the addition of potassium *tert*-butoxide (37 mg, 0.33 mmol), which turned the reaction from clear to a burnt orange color. The vial was then backfilled with inert gas, turning the reaction mixture a bright pink color. The reaction was stirred at 20 °C for 30 minutes before quenching with water. After quenching was complete, the crude mixture was diluted with EtOAc before being washed with water and brine. The organic layer was then dried over anhydrous $MgSO_4$ and concentrated under vacuum to afford the crude material, a pink oil. This was then purified by normal phase silica gel chromatography, and fractions of interest were combined before drying under vacuum to afford the title compound as a pink solid, (38.2 mg, yield 52%, purity >99%); **^{1}H NMR** (400 MHz, DMSO-d_6) δ 9.02 (s, 1H), 7.29 (dd, J = 1.38, 8.38 Hz, 1H), 7.22 (d, J = 1.63 Hz, 1H), 7.01 (dt, J = 1.38, 7.63 Hz, 1H), 6.92 (dd, J = 1.38, 7.75 Hz, 1H), 6.77 - 6.82 (m, 1H), 6.75 (d, J = 8.25 Hz, 1H), 6.68 (dd, J = 1.06, 7.94 Hz, 1H). **LCMS** $[M-H]^-$ 266.

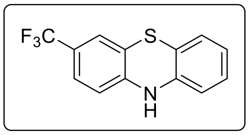

2-((2-Bromophenyl)thio)-5-methoxyaniline (12): To a reaction vial containing **7** (190 mg, 1.0 mmol) and **11** (190 mg, 1.1 mmol) was added DMF (4 mL). Cesium carbonate (650 mg, 2.0 mmol) was then added to the vial and stirred at 80 °C for 1 hour. After 1 hour, the mixture was cooled and diluted with EtOAc (50 mL) and washed with water and brine. The organic layer was then concentrated under reduced pressure to afford the crude as brown liquid. The crude was then dissolved in anhydrous EtOAc and Sn(II)Cl_2 (1.90 g, 10. mmol) was added in portion wise manner. The resulting mixture was then stirred at room temperature for 24 hours. After completion, water was added, and the organic layer was separated and washed with brine solution. It was then dried over anhydrous $MgSO_4$ and concentrated under vacuum to afford the crude. The crude was then purified by normal phase chromatography (5-30% EtOAc/Hexane), and fractions of interest were combined before drying under vacuum to afford the title compound as a brown gum, (158 mg, yield 50%, purity 92%). **^{1}H NMR** (400 MHz, $CDCl_3$) δ 7.49 (d, J = 7.8 Hz, 1H), 7.36 (d, J = 8.4

Hz, 1H), 7.09 (t, J = 7.6 Hz, 1H), 6.98 – 6.89 (m, 1H), 6.61 (dd, J = 8.0, 1.1 Hz, 1H), 6.37 (dt, J = 6.4, 2.5 Hz, 2H), 4.31 (s, 2H), 3.81 (s, 3H). **LCMS** $[M+H]^+$ 311.

2-Methoxy-10*H*-phenthiazine (2h): In a reaction vial, **12** (190 mg, 0.60 mmol), sodium *tert*-butoxide (120 mg, 1.2 mmol) and dppf (33 mg, 0.060 mmol) were dissolved in anhydrous toluene (4.0 mL). The resulting mixture was degassed with N_2 for 10 min and then $Pd_2(dba)_3$ (55 mg, 0.060 mmol) was added to it. The resulting mixture was then heated to 110 °C for 24 hours. After completion, the reaction mixture was cooled to room temperature and passed through a Celite plug and washed with EtOAc. Volatiles were then removed under reduced pressure to afford the crude which was then purified by normal phase chromatography (5-30% EtOAc/Hexane), and fractions of interest were combined before drying under vacuum to afford **2h** as light brown solid, (30 mg, yield 20%, purity 94%); **^{1}H NMR** (400 MHz, DMSO-d_6) δ 8.59 (s, 1H), 6.98 (t, J = 7.5 Hz, 1H), 6.91 (d, J = 7.4 Hz, 1H), 6.82 (d, J = 8.4 Hz, 1H), 6.75 (t, J = 7.4 Hz, 1H), 6.68 (d, J = 7.8 Hz, 1H), 6.38 (d, J = 8.3 Hz, 1H), 6.33 (d, J = 2.4 Hz, 1H), 3.68 (s, 3H). **LCMS** $[M+H]^+$ 230.

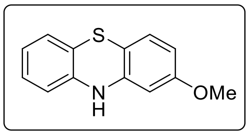

General procedure for the synthesis of 2k-l: To a stirred solution of 2-amino-4-(trifluoromethyl)benzenethiol (1.0 eq.) and 4-alkyl substituted cyclohexanone (1.2 eq.) in DMSO (5 mL) was added cesium carbonate (2.0 eq.) and heated to 110 °C for 48 hours under air. After reaction completion, it was cooled to room temperature, and water was added to it. Then it was extracted using EtOAc (2x100 mL). The combined organic layer was then dried over anhydrous Na_2SO_4 and concentrated to 30-40 mL under reduced pressure to afford the crude material. The crude was then purified by flash chromatography (0-40% EtOAc in hexane) to afford the title compound.

7-Methyl-2-(trifluoromethyl)-10*H*-phenthiazine (2k): Using the general procedure, 2-amino-4-(trifluoromethyl)benzenethiol (580 mg, 3.0 mmol) and 4-methylcyclohexanone (410 mg, 3.6 mmol) were reacted to afford **2k** as an off-white solid, (318 mg, yield 38%, purity 89%); **^{1}H NMR** (400 MHz, DMSO-d_6) δ 8.74 (s, 1H), 7.05 (dd, J = 34.8, 8.0 Hz, 2H), 6.88 (s, 1H), 6.82 (d, J = 8.0 Hz, 1H), 6.76 (s, 1H), 6.55 (d, J = 8.0 Hz, 1H), 2.13 (s, 3H); **^{19}F NMR** (376 MHz, DMSO) δ -61.69 (s). **LCMS** $[M+H]^+$ 282.

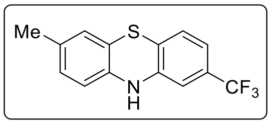

7-(*Tert*-butyl)-2-(trifluoromethyl)-10*H*-phenthiazine (2l): Using the general procedure, 2-amino-4-(trifluoromethyl)benzenethiol (580 mg, 3.0 mmol) and 4-methylcyclohexanone (550 mg, 3.6 mmol) were reacted to afford **2l** as an off-white solid, (290 mg, yield 30%, purity 91%); **^{1}H NMR** (400 MHz, DMSO-d_6) δ 8.78 (s, 1H), 7.10 (d, J = 8.0 Hz, 1H), 7.03 (dd, J = 13.6, 5.3 Hz, 2H), 6.94 – 6.87 (m, 2H), 6.60 (d, J = 8.3 Hz, 1H), 1.20 (s, 9H); **^{19}F NMR** (376 MHz, DMSO) δ -61.67 (s). **LCMS** $[M+H]^+$ 324.

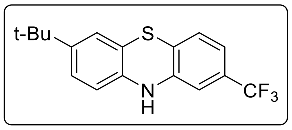

General procedure for the synthesis of 2m-o: To a heat-dried reaction vial, a substituted 2-aminobenzenethiol, **15** (1.0 eq.), 3-bromo-2-chloropyridine, **16** (1.0 eq.), and cesium carbonate (2.0 eq.) were dissolved in anhydrous DMSO (2 mL). After addition of all reagents, the vial was backfilled with inert gas. The reaction was heated to 120 °C for 16 hours. After reaction completion, it was cooled to room temperature and quenched with water. Then it was extracted using EtOAc, and the combined organic layer was washed with saturated brine solution and then dried over $MgSO_4$. Crude material was then dissolved in 2-3 mL DCM and purified by normal phase chromatography (0-100% EtOAc/Hexane), and fractions of interest were combined before drying under vacuum to afford the title compound.

10H-Benzo[b]pyrido[2,3-e][1,4]thiazine (2m): The title compound (Cat. No A509943-1g, CAS No. 261-96-1) was purchased from Ambeed and used for subsequent reactions.

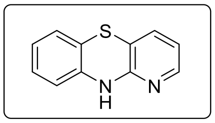

3-(Trifluoromethyl)-10H-benzo[b]pyrido[2,3-e][1,4]thiazine (2n): Using the general procedure, 2-aminobenzenethiol (0.43 mL, 4.0 mmol) and 3-bromo-2-chloro-5-(trifluoromethyl)pyridine (1.0 g, 4.0 mmol) were reacted to afford **2n** as a yellow solid, (712 mg, yield 64%, purity 95%); **^{1}H NMR** (400 MHz, DMSO-d_6) δ 9.77 (s, 1H), 8.11 (s, 1H), 7.61 (s, 1H), 6.99 - 7.05 (m, 1H), 6.93 (d, J = 7.34 Hz, 1H), 6.80 - 6.86 (m, 2H). **LCMS** $[M+H]^+$ 269.

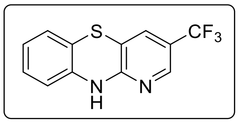

7-Chloro-3-(trifluoromethyl)-10H-benzo[b]pyrido[2,3-e][1,4]thiazine (2o): Using the general procedure, 2-amino-5-chlorobenzenethiol (100 mg, 0.63 mmol) and 3-bromo-2-chloro-5-(trifluoromethyl)pyridine (160 mg, 0.63 mmol) were reacted to afford **2o** as a brown solid, (174 mg, yield 91%, purity >99%);

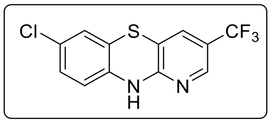

^{1}H NMR (400 MHz, DMSO-d_6) δ 9.88 (s, 1H), 8.11 - 8.13 (m, 1H), 7.65 (d, J = 1.96 Hz, 1H), 7.07 - 7.09 (m, 1H), 7.05 - 7.06 (m, 1H), 6.79 (d, J = 8.07 Hz, 1H). **LCMS** $[M+H]^+$ 303.

General procedure for the synthesis of 21a-g: Step 1: To a reaction vial, aniline, **2a-g** (1.0 eq.) was dissolved in anhydrous DMF (5 mL) before cooling to 0 °C. Then NaH (60% dispersion in mineral oil, 3.0 eq.) was added slowly before letting the reaction warm to room temperature. The reaction sat undisturbed for 30 minutes before cooling back to 0 °C. Another vial filled with 1,4-bis(bromomethyl)benzene, **17** (3.0 eq.) was prepared and dissolved in DMF (5 mL). The vial with the aniline sodium salt was pipetted and added to the cooled vial filled with **17** slowly and dropwise. After the full addition, the vial was warmed to room temperature and left to stir for 2 hours. After reaction completion, it was quenched with sodium bicarbonate, extracted using EtOAc, and the combined organic layer was dried over $MgSO_4$. Crude material was then purified by normal phase chromatography (0-100% EtOAc/Hexane), and fractions of interest were combined before drying under vacuum to afford a mixture of **18a-g** and **19a-g** which were carried onto the next step. Step 2: To a microwave reaction vial, impure **18a-g** (1.0 eq.), potassium phosphate (2.0 eq.), and potassium iodide (0.10 eq.) were dissolved in anhydrous DMF (2 mL). Then 2-(piperazin-1-yl)ethan-1-ol, **20** (2.0 eq.), was added. The reaction was heated to 80 °C for 2 hours. Once complete, the reaction was diluted with EtOAc. The organic layer was washed with 1N HCl, and the pH was then adjusted to 8. Again, this layer was washed with EtOAc and was dried over $MgSO_4$, filtered, and concentrated under vacuum. The resulting crude material was then purified by reverse phase chromatography RediSep Rf Gold pre-packed C18 cartridges with a 5 to 100% acetonitrile/water/0.1% formic acid gradient. Fractions were then lyophilized. Upon isolation, each compound was dissolved in methanol and treated with 4M HCl in dioxane (4-5 drops). The solution was stirred in a vial at room temperature for 30-60 minutes, followed by liquids being evaporated off to afford the title compound.

2-(4-(4-((10H-Phenothiazin-10-yl)methyl)benzyl)piperazin-1-yl)ethan-1-ol hydrochloride

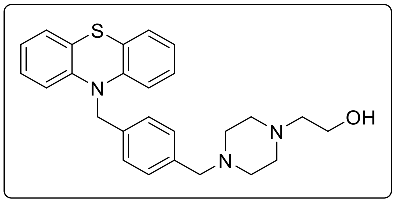

(21a): Using the general procedure, **2a** (1.0 g, 3.8 mmol) was transformed to **21a** as a green solid, (93 mg, yield 21%, purity 95%); **^{1}H NMR** (400 MHz, Methanol-d_4) δ 7.50 – 7.58 (m, 4H), 7.10 (d, J = 7.34 Hz, 2H), 7.01 (br. s., 2H), 6.88 (br. s., 2H), 6.77 (d, J = 6.36 Hz, 2H), 5.18 (br. s., 2H), 4.42 (s, 2H), 3.91 (d, J = 3.18 Hz, 2H), 3.61 (br. s., 8H),

3.40 (br. s., 2H). **LCMS** $[M+H]^+$ 432; **HRMS** $[M+H]^+$ Calcd for $C_{26}H_{29}N_3OS$ 432.2109; found 432.2094.

2-(4-(4-((10H-Phenoxazin-10-yl)methyl)benzyl)piperazin-1-yl)ethan-1-ol hydrochloride (21b):

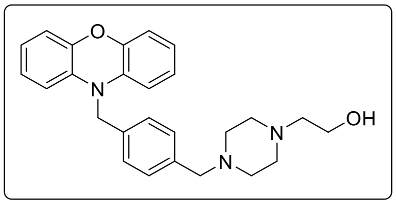

Using the general procedure, **2b** (500 mg, 2.7 mmol) was transformed to **21b** as a green solid, (39 mg, yield 2%, purity >99%); **^{1}H NMR** (400 MHz, Methanol-d_4) δ 7.59 (d, J = 8.07 Hz, 2H), 7.47 (d, J = 7.82 Hz, 2H), 6.67 (br. s., 6H), 6.39 (br. s., 2H), 4.89 – 5.14 (m, 2H), 3.89 – 3.94 (m, 2H), 3.47 - 3.86 (m, 8H), 3.41 (br. s., 2H). **LCMS** $[M+H]^+$ 416; **HRMS** $[M+H]^+$ Calcd for $C_{26}H_{29}N_3O_2$ 416.2337; found 416.2332.

2-(4-(4-((9H-Carbazol-9-yl)methyl)benzyl)piperazin-1-yl)ethan-1-ol hydrochloride (21c):

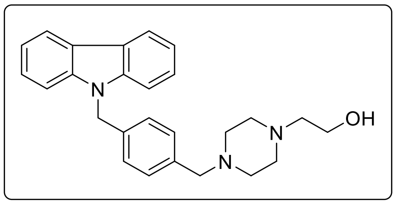

Using the general procedure, **2c** (500 mg, 3.0 mmol) was transformed to **21c** as a tan solid, (18 mg, yield 9%, purity 98%); **^{1}H NMR** (400 MHz, Methanol-d_4) δ 8.12 (dd, J = 2.08, 7.70 Hz, 2H), 7.43 – 7.48 (m, 4H), 7.37 – 7.43 (m, 2H), 7.23 – 7.27 (m, 2H), 7.19 – 7.23 (m, 2H), 5.64 (d, J = 2.45 Hz, 2H), 4.26 (br. s., 2H), 3.84 – 3.89 (m, 2H), 3.61 (br. s., 4H), 3.41 (br. s., 4H), 3.32 – 3.36 (m, 2H). **LCMS** $[M+H]^+$ 400; **HRMS** $[M+H]^+$ Calcd for $C_{26}H_{29}N_3O$ 400.2389; found 400.2382.

2-(4-(4-((Diphenylamino)methyl)benzyl)piperazin-1-yl)ethan-1-ol hydrochloride (21d):

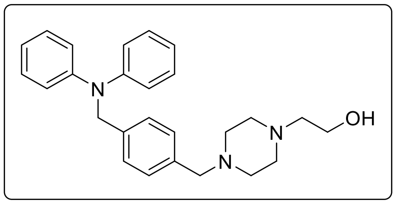

Using the general procedure, **2d** (500 mg, 3.0 mmol) was transformed to **21d** as a tan solid, (51 mg, yield 6%, purity 97%); **^{1}H NMR** (400 MHz, Methanol-d_4) δ 7.44 – 7.55 (m, 4H), 7.17 – 7.25 (m, 4H), 7.00 – 7.07 (m, 4H), 6.87 – 6.94 (m, 2H), 5.05 (s, 2H), 4.30 (br. s., 2H), 3.89 (dd, J = 4.16, 5.87 Hz, 2H), 3.37 – 3.85 (m, 8H), 3.34 (br. s., 2H). **LCMS** $[M+H]^+$ 402; **HRMS** $[M+H]^+$ Calcd for $C_{26}H_{31}N_3O$ 402.2545; found 402.2532.

2-(4-(4-((10,11-Dihydro-5H-dibenzo[b,f]azepin-5-yl)methyl)benzyl)piperazin-1-yl)ethan-1-ol hydrochloride (21e): Using the general procedure, **2e** (500 mg, 3.0 mmol) was transformed to **21e** as a tan solid, (88 mg, yield 9%, purity 98%); **^{1}H NMR** (400 MHz, Methanol-d_4) δ 7.58 (d, J = 7.82 Hz, 2H), 7.48 (d, J = 8.07 Hz, 2H), 7.14 (d, J = 7.82 Hz, 2H), 7.07 (dd, J = 1.47, 7.58 Hz, 2H), 7.03 (dt, J = 1.47, 7.70 Hz, 2H), 6.86 (dt, J = 0.98, 7.34 Hz, 2H) 5.00 (s, 2H), 4.36 (s, 2H), 3.88 – 3.92 (m, 2H), 3.48 – 3.80 (m, 8H), 3.36 – 3.41 (m, 2H), 3.22 (s, 4H). **LCMS** $[M+H]^+$ 428; **HRMS** $[M+H]^+$ Calcd for $C_{28}H_{33}N_3O$ 428.2702; found 428.2688.

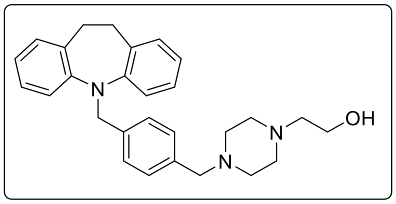

2-(4-(4-((2-Chloro-10H-phenothiazin-10-yl)methyl)benzyl)piperazin-1-yl)ethan-1-ol hydrochloride (21f): Using the general procedure, **2f** (500 mg, 2.1 mmol) was transformed to **21f** as a dark-colored solid, (3.6 mg, yield 37%, purity >99%); **^{1}H NMR** (400 MHz, Methanol-d_4) δ 7.49 – 7.59 (m, 4H), 7.11 (d, J = 7.58 Hz, 1H), 7.06 (d, J = 8.07 Hz, 1H), 7.01 – 7.04 (m, 1H), 6.87 – 6.94 (m, 2H), 6.79 (d, J = 7.82 Hz, 1H), 6.74 (d, J = 1.71 Hz, 1H), 5.16 (br. s., 2H), 4.39 (br. s., 2H), 3.91 (br. s., 2H) 3.47 – 3.79 (m, 8H), 3.39 (br. s., 2H). **LCMS** $[M+H]^+$ 466; **HRMS** $[M+H]^+$ Calcd for $C_{26}H_{28}ClN_3OS$ 466.1720; found 466.1710.

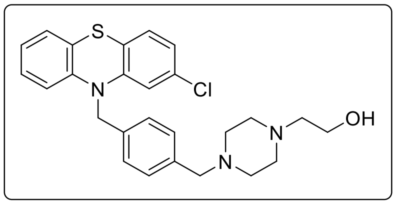

10-(4-((4-(2-Hydroxyethyl)piperazin-1-yl)methyl)benzyl)-10H-phenothiazine-2-carbonitrile hydrochloride (21g): Using the general procedure, **2g** (100 mg, 0.45 mmol) was transformed to **21g** as a yellow solid, (18 mg, yield 5%, purity 97%); **^{1}H NMR** (400 MHz, DMSO-d_6) δ 7.57 (br. s., 2H), 7.40 (d, J = 7.34 Hz, 2H), 7.35 (s, 2H), 7.13 - 7.19 (m, 2H), 7.11 (t, J = 7.34 Hz, 1H), 6.93 - 6.99 (m, 1H), 6.82 (d, J = 8.31 Hz, 1H), 5.21 (s, 1H), 5.21 (s, 2H), 3.76 (br. s., 2H), 3.69 (dd, J = 5.26, 14.31 Hz, 2H), 3.48 (dd, J, = 4.40, 11.25 Hz, 8H), 3.20 (br. s., 2H). **LCMS** $[M+H]^+$ 457; **HRMS** $[M+H]^+$ Calcd for $C_{27}H_{28}N_4OS$ 457.2062; found 457.2108.

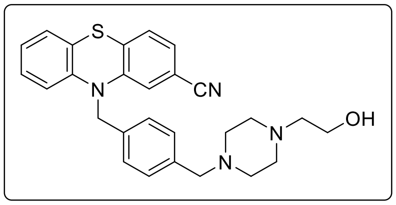

1-(2-((*Tert*-butyldimethylsilyl)oxy)ethyl)-4-(4-(chloromethyl)benzyl)piperazine (22): The TBS-protected alcohols were synthesized by the modified literature procedure from Elmabruk et. al.[34] To a stirred solution of 1-piperazine ethanol (1.3 g, 10 mmol) in anhydrous THF (2 mL/mmol)

was added 1*H*-imidazole (1.0 g, 15 mmol) at 0 °C and stirred for 10 min. Then a solution of TBSCl

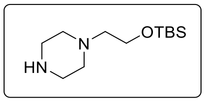

(2.3 g, 15 mmol) in anhydrous THF was added slowly at 0 °C over 15 min and the reaction was allowed to warm up to room temperature. After 16 hours, the reaction was quenched by the slow addition of water under ice-cooling and extracted with 2-propanol: DCM mixture (1:3, 3x100 mL). The combined layer was then washed with brine solution, dried over anhydrous Na_2SO_4, and concentrated under reduced pressure to afford the crude as a colorless gummy liquid. It was then dissolved in heptane (20 ml) and heated to 60 °C for 1 hour and then cooled to 0 °C. Precipitation of white solid was observed. The solid was then filtered and washed with cold hexane to afford the title compound as a white solid (1.7 g, yield 69%, purity not determined - not UV active); **^{1}H NMR** (400 MHz, $CDCl_3$) δ 3.68 (t, *J* = 5.5 Hz, 2H), 3.22 – 3.03 (m, 4H), 2.78 (d, *J* = 4.2 Hz, 4H), 2.53 (t, *J* = 5.5 Hz, 2H), 0.83 (s, 9H), -0.00 (s, 6H).

1-(2-((*Tert*-butyldimethylsilyl)oxy)ethyl)-4-(4-(chloromethyl)benzyl)piperazine (24): To a

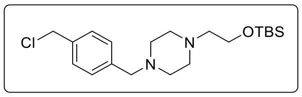

stirred solution of bis(chloromethyl)benzene derivative (530 mg, 3.0 mmol) and TBS-protected alcohol (370 mg, 1.5 mmol) in anhydrous acetonitrile was added DIEA (0.52 mL, 3.0 mmol) at room temperature and stirred vigorously for 16 hours. Then acetonitrile was removed under reduced pressure to afford the crude as a gummy liquid. Unless until mentioned, the crude was then purified by normal phase flash chromatography (0-10% MeOH/EtOAc) to afford 1-(2-((*tert*-butyldimethylsilyl)oxy)ethyl)-4-(4-(chloromethyl)benzyl)piperazine as gummy liquid (265 mg, yield 46%, purity 85%); **^{1}H NMR** (400 MHz, $CDCl_3$) δ 7.37 – 7.28 (m, 4H), 4.57 (s, 2H), 3.74 (t, *J* = 6.5 Hz, 2H), 3.49 (s, 2H), 2.51 (dd, *J* = 17.9, 11.4 Hz, 10H), 0.88 (s, 9H), 0.05 (s, 6H).

General procedure for the synthesis of 21h-o: To a heat-dried reaction vial, a stirred solution of PTZ derivative **2h**-**2o**, (1.0 eq.) in anhydrous DMF (1-2 mL) was added NaH (60% dispersion in mineral oil, 3.0 eq.) at 0 °C under inert atmosphere and stirred for 15-20 min. Then a solution of the benzyl chloride derivative **24** (1.5 eq.) in anhydrous DMF (1 mL) was added to the reaction mixture at 0 °C and stirred for 5 min at room temperature. The reaction mixture was then heated to 60 °C and stirred for 2-3 hours. After completion of the reaction (monitored by LCMS), the reaction was cooled to 0 °C and quenched with the addition of ice water and extracted with ethyl acetate (2x50 mL). The combined organic layer was dried over anhydrous $MgSO_4$, filtered, and

concentrated under reduced pressure to afford the crude as a gummy liquid. It was then dissolved in anhydrous THF and was added a solution of TBAF in THF (1M solution, 10. eq.). The reaction mixture was then stirred at room temperature for 1 hour. After completion, it was quenched with the addition of a saturated solution of $NaHCO_3$ and extracted with ethyl acetate (2x50 mL). The combined organic layer was dried over anhydrous $MgSO_4$, filtered, and concentrated under reduced pressure to afford the crude as a gummy liquid. It was then purified by reverse phase chromatography RediSep Rf Gold pre-packed C18 cartridges with a 5 to 100% acetonitrile/water/0.1% formic acid gradient. Fractions were then lyophilized to afford the title compound.

2-(4-(4-((2-Methoxy-10H-phenothiazin-10-yl)methyl)benzyl)piperazin-1-yl)ethan-1-ol

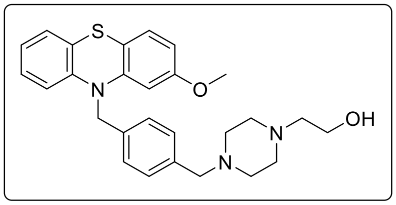

(21h): Using the general procedure, **2h** (750 mg, 3.3 mmol) was transformed into **21h** as a white solid, (25 mg, yield 12%, purity 96%); **^{1}H NMR** (400 MHz, DMSO-d_6) δ 7.25 (d, J = 5.38 Hz, 4H), 7.11 - 7.15 (m, 1H), 7.06 (s, 1H), 7.02 (d, J = 8.56 Hz, 1H), 6.90 (s, 1H), 6.80 (d, J = 8.31 Hz, 1H), 6.52 (s, 1H), 6.35 (d, J = 2.45 Hz, 1H), 5.10 (s, 2H), 4.30 - 4.37 (m, 1H), 3.59 (s, 3H), 3.46 (d, J = 5.87 Hz, 2H), 3.39 (s, 2H), 2.35 (br. s., 8H). Note that N$\underline{CH_2}$$CH_2$OH is not observed due to overlap with the H_2O peak. **LCMS** $[M+H]^+$ 462; **HRMS** $[M+H]^+$ Calcd for $C_{27}H_{31}N_3O_2S$ 462.2215; found 462.2221.

2-(4-(4-((3-Methyl-10H-phenothiazin-10-yl)methyl)benzyl)piperazin-1-yl)ethan-1-ol (21i):

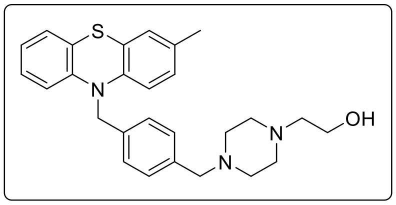

Using the general procedure, **2i** (50 mg, 0.24 mmol) and **24** (135 mg, 0.35 mmol) were reacted to afford **21i** as a peach-colored solid, (23 mg, yield 21%, purity 96%); **^{1}H NMR** (400 MHz, DMSO-d_6) δ 7.22 (br. s., 4H), 7.11 (d, J = 7.58 Hz, 1H), 7.04 (t, J = 7.58 Hz, 1H), 6.94 (br. s., 1H), 6.86 (dd, J = 7.58, 13.94 Hz, 2H), 6.73 (d, J = 7.83 Hz, 1H), 6.64 (d, J = 8.07 Hz, 1H), 5.06 (br. s., 2H), 4.34 (br. s., 1H), 3.45 (br. s., 2H), 3.38 (br. s., 2H), 2.52 – 2.56 (m, 2H), 2.27- 2.41 (m, 8H), 2.15 (s, 3H). **LCMS** $[M+H]^+$ 446; **HRMS** $[M+H]^+$ Calcd for $C_{27}H_{31}N_3OS$ 446.2266; found 446.2265.

2-(4-(4-((3-(Trifluoromethyl)-10H-phenothiazin-10-yl)methyl)benzyl)piperazin-1-yl)ethan-1-ol (21j): Using the general procedure, **2j** (100 mg, 0.37 mmol) and **24** (220 mg, 0.56 mmol) were reacted to afford **21j** as a yellow solid, (42 mg, yield 23%, purity 99%); ^{1}**H NMR** (400 MHz, DMSO-d_6) δ 7.42 (s, 1H), 7.36 (d, *J* = 8.56 Hz, 1H), 7.21 (s, 4H), 7.11 (d, *J* = 7.34 Hz, 1H), 7.02 - 7.08 (m, 1H), 6.88 - 6.94 (m, 1H), 6.82 (d, *J* = 8.56 Hz, 1H), 6.76 (d, *J* = 8.31 Hz, 1H), 5.12 (s, 2H), 3.45 (t, *J* = 6.11 Hz, 2H), 3.37 (s, 2H), 2.45 – 2.47 (m, 4H), 2.28 – 2.43 (m, 8H). **LCMS** $[M+H]^+$ 500; **HRMS** $[M+H]^+$ Calcd for $C_{27}H_{28}F_3N_3OS$ 500.1983; found 500.1980.

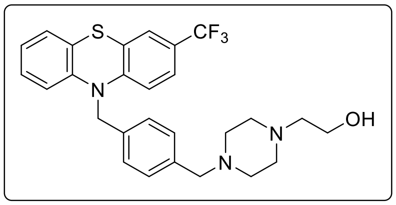

2-{4-[(*p*-{[7-Methyl-2-(trifluoromethyl)-10-phenothiazinyl]methyl}phenyl)methyl]-1-piperazinyl}ethanol (**21k)** Using the general procedure, **2k** (17 mg, 0.058 mmol) was reacted with **24** (33.3 mg, 0.087 mmol) to afford the title compound as white solid (17 mg, yield 57%, purity 97%). ^{1}**H NMR** (400 MHz, DMSO-d_6) *δ* 7.33 (d, *J* = 7.9 Hz, 1H), 7.25 (q, *J* = 8.3 Hz, 4H), 7.19 (d, *J* = 8.4 Hz, 1H), 7.00 (s, 1H), 6.92 (d, *J* = 5.1 Hz, 2H), 6.76 (d, *J* = 8.3 Hz, 1H), 5.14 (s, 2H), 4.33 (t, *J* = 5.4 Hz, 1H), 3.46 (q, *J* = 6.2 Hz, 2H), 3.39 (s, 2H), 2.35 (t, *J* = 6.4 Hz, 10H), 2.18 (s, 3H); ^{19}**F NMR** (376 MHz, DMSO-d_6) δ -61.35 (s). **HRMS** $[M+H]^+$ Calcd for $C_{28}H_{31}F_3N_3OS$ 514.3124; found 514.3124.

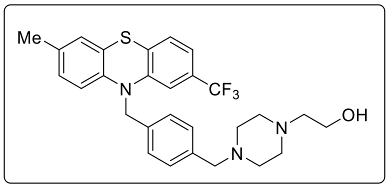

2-{4-[(*p*-{[7-(*Tert*-butyl)-2-(trifluoromethyl)-10-phenothiazinyl]methyl}phenyl)methyl]-1-piperazinyl}ethanol (21l): Using the general procedure, **2l** (29 mg, 0.087 mmol) was reacted with **24** (50 mg, 0.088 mmol) to afford the title compound as white solid (18 mg, yield 37%, purity 98%). ^{1}**H NMR** (400 MHz, DMSO-d_6) *δ* 7.33 (d, *J* = 8.0 Hz, 1H), 7.26 (q, *J* = 8.1 Hz, 4H), 7.22 – 7.11 (m, 3H), 6.91 (s, 1H), 6.80 (d, *J* = 8.4 Hz, 1H), 5.15 (s, 2H), 4.33 (t, *J* = 5.2 Hz, 1H), 3.46 (q, *J* = 6.0 Hz, 2H), 3.39 (s, 2H), 2.49 – 2.22 (m, 10H), 1.22 (s, 9H); ^{19}**F NMR** (376 MHz, DMSO-d_6) δ -61.36 (s). **HRMS** $[M+H]^+$ Calcd for $C_{31}H_{37}F_3N_3OS$ 556.2604; found 556.2593.

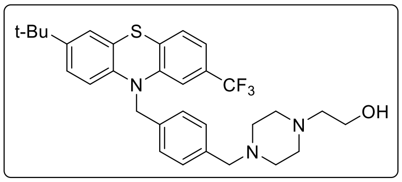

2-(4-(4-((10H-Benzo[b]pyrido[2,3-e][1,4]thiazin-10-yl)methyl)benzyl)piperazin-1-yl)ethan-1-ol (21m): Using the general procedure, **2m** (200 mg, 1.0 mmol) and **24** (580 mg, 1.5 mmol) were reacted to afford **21m** as a light yellow solid, (71 mg, yield 15%, purity 95%); **^{1}H NMR** (400 MHz, DMSO-d_6) δ 7.94 (dd, J = 1.22, 4.89 Hz, 1H), 7.46 (dd, J = 1.22, 7.58 Hz, 1H), 7.13 - 7.26 (m, 4H), 7.08 (d, J = 6.60 Hz, 1H), 6.98 - 7.03 (m, 1H), 6.85 - 6.90 (m, 2H), 6.72 (d, J = 8.31 Hz, 1H), 5.30 (s, 2H), 3.51 (br. s., 2H), 3.46 (t, J = 6.24 Hz, 2H), 3.38 (s, 2H), 2.36 (t, J = 6.36 Hz, 8H). **LCMS** $[M+H]^+$ 433; **HRMS** $[M+H]^+$ Calcd for $C_{25}H_{28}N_4OS$ 433.2062; found 433.2063.

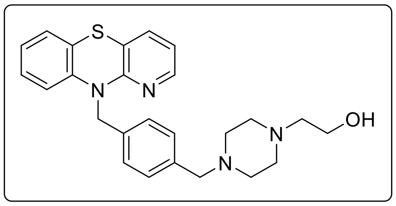

2-(4-(4-((3-(Trifluoromethyl)-10H-benzo[b]pyrido[2,3-e][1,4]thiazin-10-yl)methyl)benzyl)piperazin-1-yl)ethan-1-ol (21n): Using the general procedure, **2n** (100 mg, 0.37 mmol) and **24** (220 mg, 0.56mmol) were reacted to afford **21n** as a light yellow solid, (72 mg, yield 40%, purity 99%); **^{1}H NMR** (400 MHz, DMSO-d_6) δ 8.26 (s, 1H), 7.82 (s, 1H), 7.17 - 7.23 (m, 4H), 7.10 (d, J = 7.34 Hz, 1H), 7.01 - 7.07 (m, 1H), 6.91 - 6.96 (m, 1H), 6.75 (d, J = 8.07 Hz, 1H), 5.33 (br. s., 2H), 4.38 (br. s., 1H), 3.46 (t, J = 6.24 Hz, 2H), 3.38 (s, 2H), 2.27 - 2.43 (m, 8H). Note that N$\underline{CH_2}$$CH_2$OH is not observed due to overlap with other peaks. **LCMS** $[M+H]^+$ 501; **HRMS** $[M+H]^+$ Calcd for $C_{26}H_{27}F_3N_4OS$ 501.1936; found 501.1936.

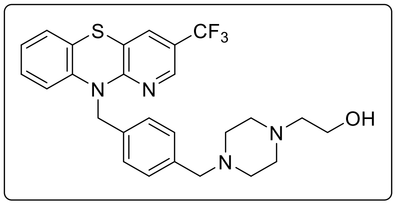

2-(4-(4-((7-Chloro-3-(trifluoromethyl)-10H-benzo[b]pyrido[2,3-e][1,4]thiazin-10-yl)methyl)benzyl)piperazin-1-yl)ethan-1-ol (21o): Using the general procedure, **2o** (150 mg, 0.50 mmol) and **24** (290 mg, 0.75 mmol) were reacted to afford **21o** as a yellow solid, (11 mg, yield 4%, purity 98%); **^{1}H NMR** (400 MHz, DMSO-d_6) δ 8.28 (s, 1H), 7.86 (d, J = 1.96 Hz, 1H), 7.25 (s, 1H), 7.17 - 7.23 (m, 4H), 7.10 (dd, J = 2.45, 8.80 Hz, 1H), 6.70 (d, J = 8.80 Hz, 1H), 5.31 (br. s., 2H), 3.46 (t, J = 6.36 Hz, 2H), 3.39 (s, 2H), 2.37 (t, J = 6.36 Hz, 8H). **LCMS** $[M+H]^+$ 535; **HRMS** $[M+H]^+$ Calcd for $C_{26}H_{26}ClF_3N_4OS$ 535.1546; found 535.1540.

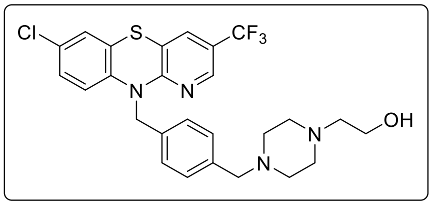

Supplementary Analysis and Compound Spectra

Acquisition Time (sec)	4.0894	***Date***	16 Sep 2021 11:44:48			***Date Stamp***	16 Sep 2021 11:44:48
File Name	H:\@Documents\NMR\SB-II-71\10\fid			***Frequency (MHz)***	400.13	***Nucleus***	1H
Number of Transients	16	***Origin***	spect	***Original Points Count***	32768	***Owner***	nmr
Points Count	32768	***Pulse Sequence***	zg30	***Receiver Gain***	645.00	***SW(cyclical) (Hz)***	8012.82
Solvent	CHLOROFORM-d			***Spectrum Offset (Hz)***	2464.9976	***Spectrum Type***	STANDARD
Sweep Width (Hz)	8012.58	***Temperature (degree C)***	25.307				

^{1}H NMR (400 MHz, CHLOROFORM-d) δ 7.24 (d, *J* = 7.83 Hz, 2H), 7.08 - 7.12 (m, 2H), 7.03 (d, *J* = 2.45 Hz, 2H), 7.01 (d, *J* = 2.45 Hz, 2H), 6.89 (t, *J* = 7.34 Hz, 1H), 5.61 (br. s., 1H), 2.32 (s, 3H)

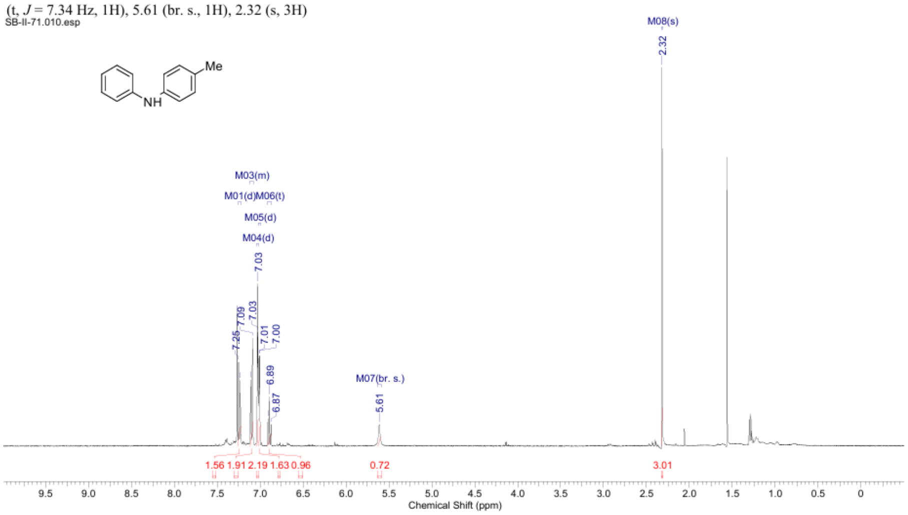

Figure S4. ^{1}H NMR of **5** in $CDCl_3$.

Acquisition Time (sec)	4.0894	***Date***	04 Nov 2021 11:32:00			***Date Stamp***	04 Nov 2021 11:32:00
File Name	H:\@Documents\NMR\SB-II-73\Pure\SB-II-73\11\fid			***Frequency (MHz)***	400.13	***Nucleus***	1H
Number of Transients	16	***Origin***	spect	***Original Points Count***	32768	***Owner***	nmr
Points Count	32768	***Pulse Sequence***	zg30	***Receiver Gain***	645.00	***SW(cyclical) (Hz)***	8012.82
Solvent	METHANOL-d4			***Spectrum Offset (Hz)***	2463.0930	***Spectrum Type***	STANDARD
Sweep Width (Hz)	8012.58	***Temperature (degree C)***	24.983				

^{1}H NMR (400 MHz, METHANOL-d_4) δ 6.93 (dt, *J* = 1.47, 7.70 Hz, 1H), 6.86 (dd, *J* = 1.34, 7.70 Hz, 1H), 6.72 - 6.78 (m, 1H), 6.69 - 6.72 (m, 2H), 6.61 (dd, *J* = 1.10, 7.95 Hz, 1H), 6.53 (d, *J* = 7.82 Hz, 1H), 2.16 (s, 3H)

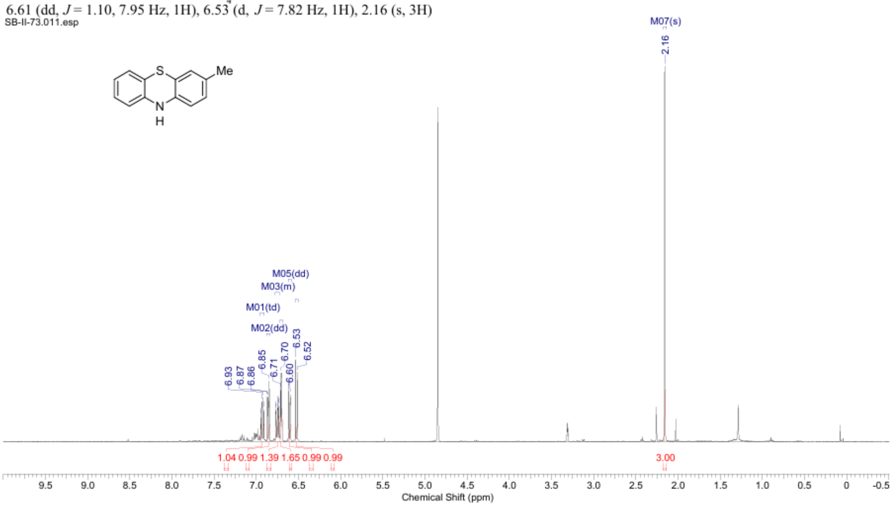

Figure S5. ^{1}H NMR of **2i** in Methanol-d_4.

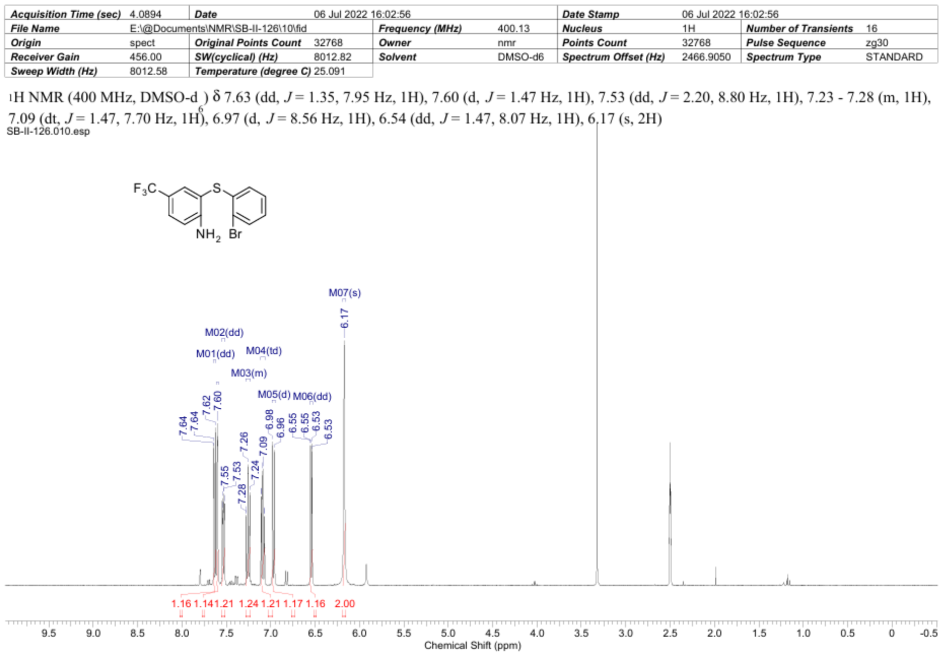

Figure S6. ^{1}H NMR of **8** in DMSO-d_6.

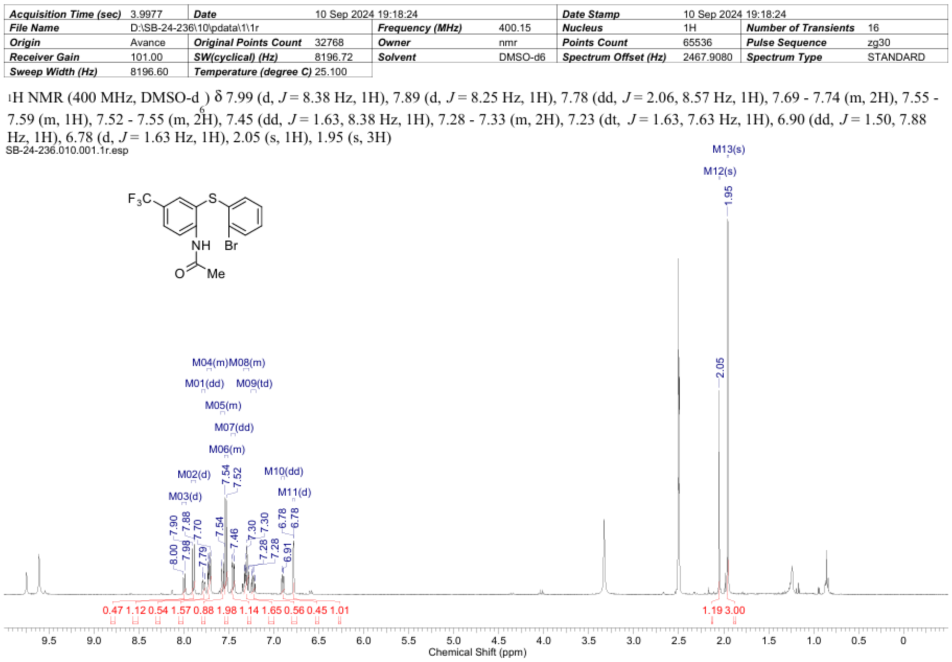

Figure S7. ^{1}H NMR of **9** in DMSO-d_6.

Acquisition Time (sec)	3.9977	Date	16 Sep 2024 20:20:16			Date Stamp	16 Sep 2024 20:20:16		
File Name	D:\SB-24-238\10\pdata\1\1r			Frequency (MHz)	400.15	Nucleus	1H	Number of Transients	16
Origin	Avance	Original Points Count	32768	Owner	nmr	Points Count	65536	Pulse Sequence	zg30
Receiver Gain	101.00	SW(cyclical) (Hz)	8196.72	Solvent	DMSO-d6	Spectrum Offset (Hz)	2466.1567	Spectrum Type	STANDARD
Sweep Width (Hz)	8196.60	Temperature (degree C)	25.100						

^{1}H NMR (400 MHz, DMSO-d_6) δ 7.98 (d, *J* = 1.38 Hz, 1H), 7.81 - 7.86 (m, 1H), 7.73 - 7.77 (m, 1H), 7.67 (dd, *J* = 1.00, 8.00 Hz, 1H), 7.59 (dd, *J* = 1.38, 7.75 Hz, 1H), 7.44 (dt, *J* = 1.50, 7.69 Hz, 1H), 7.32 - 7.37 (m, 1H), 2.16 (s, 3H)

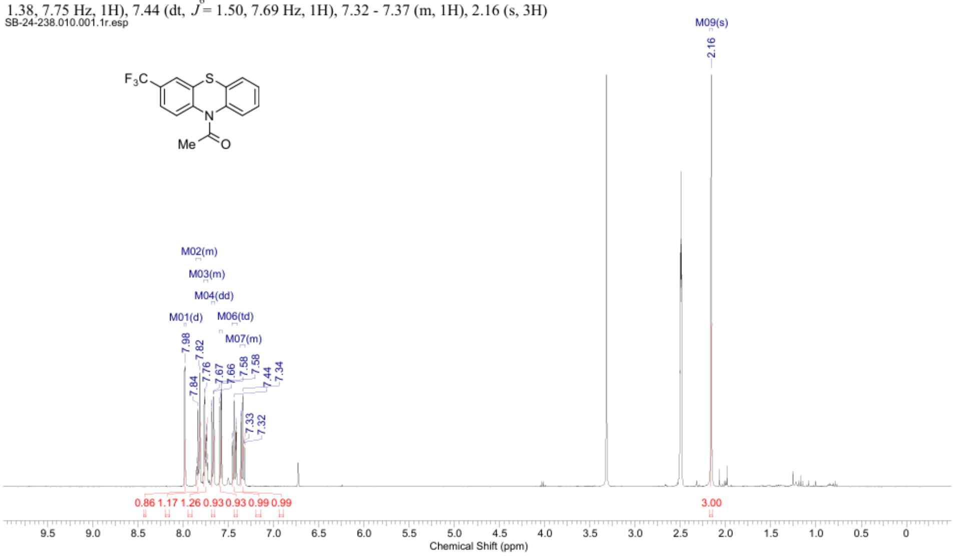

Figure S8. ^{1}H NMR of **10** in DMSO-d_6.

Acquisition Time (sec)	3.9977	Date	19 Sep 2024 18:20:48			Date Stamp	19 Sep 2024 18:20:48		
File Name	D:\SB-24-242 F8\10\pdata\1\1r			Frequency (MHz)	400.15	Nucleus	1H	Number of Transients	16
Origin	Avance	Original Points Count	32768	Owner	nmr	Points Count	65536	Pulse Sequence	zg30
Receiver Gain	101.00	SW(cyclical) (Hz)	8196.72	Solvent	DMSO-d6	Spectrum Offset (Hz)	2466.1567	Spectrum Type	STANDARD
Sweep Width (Hz)	8196.60	Temperature (degree C)	25.098						

^{1}H NMR (400 MHz, DMSO-d_6) δ 9.02 (s, 1H), 7.29 (dd, *J* = 1.38, 8.38 Hz, 1H), 7.22 (d, *J* = 1.63 Hz, 1H), 7.01 (dt, *J* = 1.38, 7.63 Hz, 1H), 6.92 (dd, *J* = 1.38, 7.75 Hz, 1H), 6.77 - 6.82 (m, 1H), 6.75 (d, *J* = 8.25 Hz, 1H), 6.68 (dd, *J* = 1.06, 7.94 Hz, 1H)

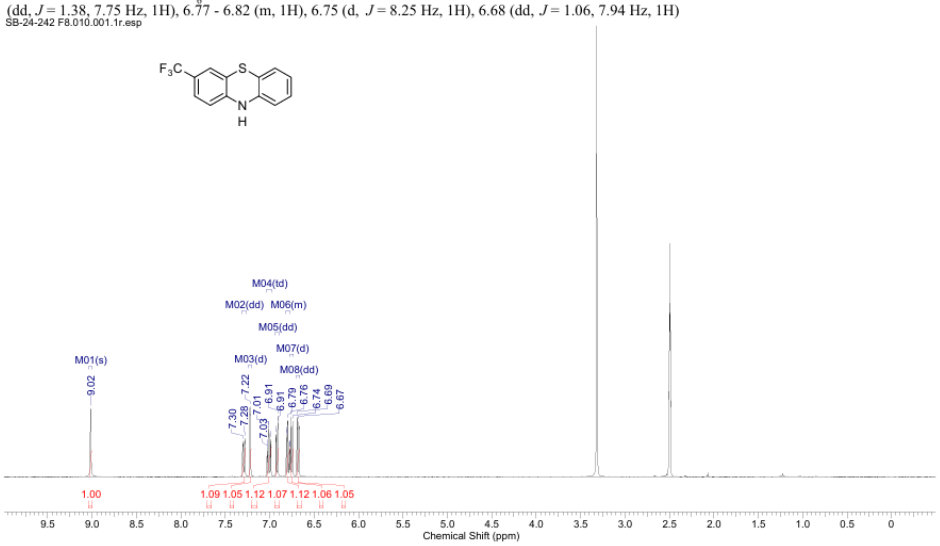

Figure S9. ^{1}H NMR of **2j** in DMSO-d_6.

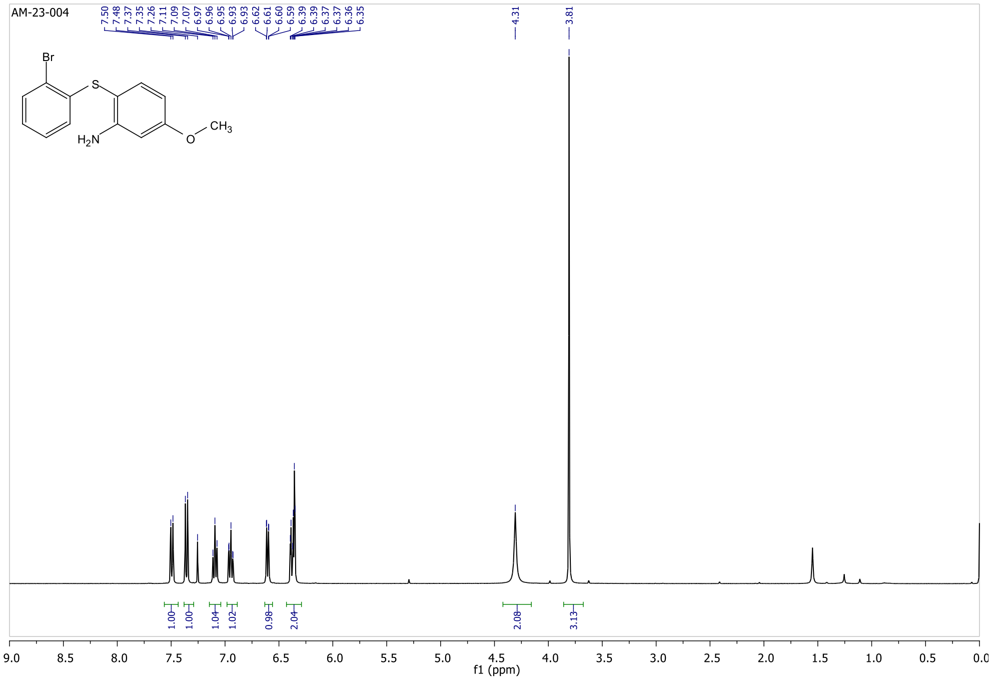

Figure S10. ^{1}H NMR of **12** in $CDCl_3$.

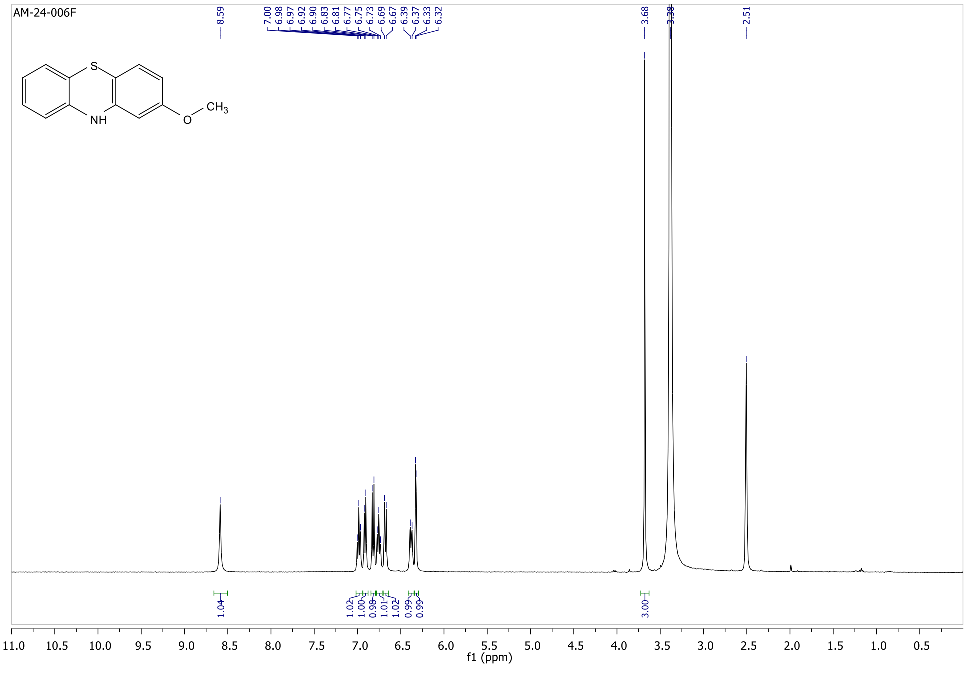

Figure S11. ^{1}H NMR of **2h** in DMSO-d_6.

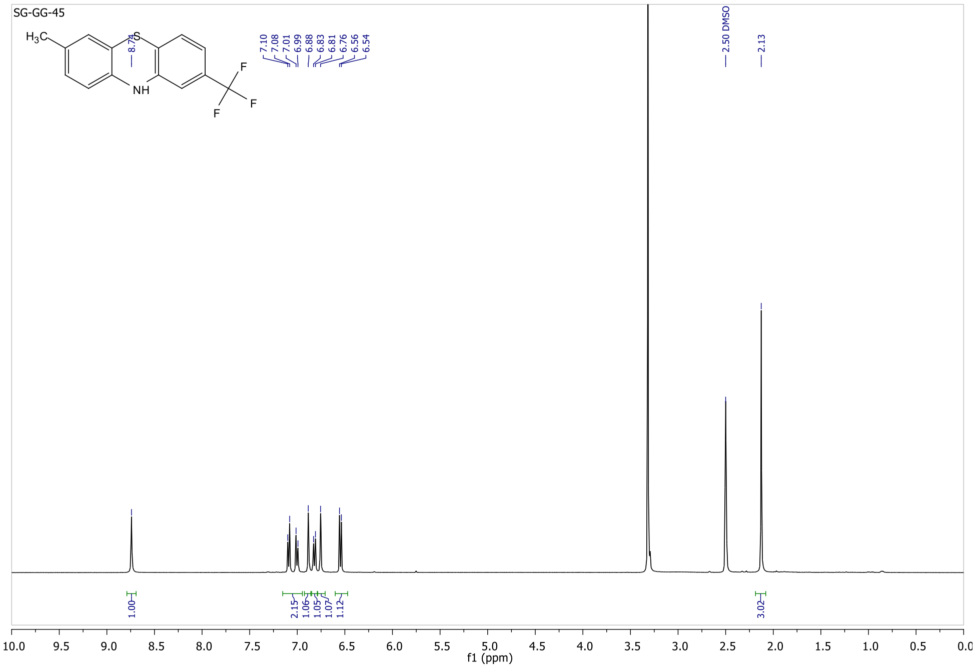

Figure S12. ^{1}H NMR of **2k** in DMSO-d_6.

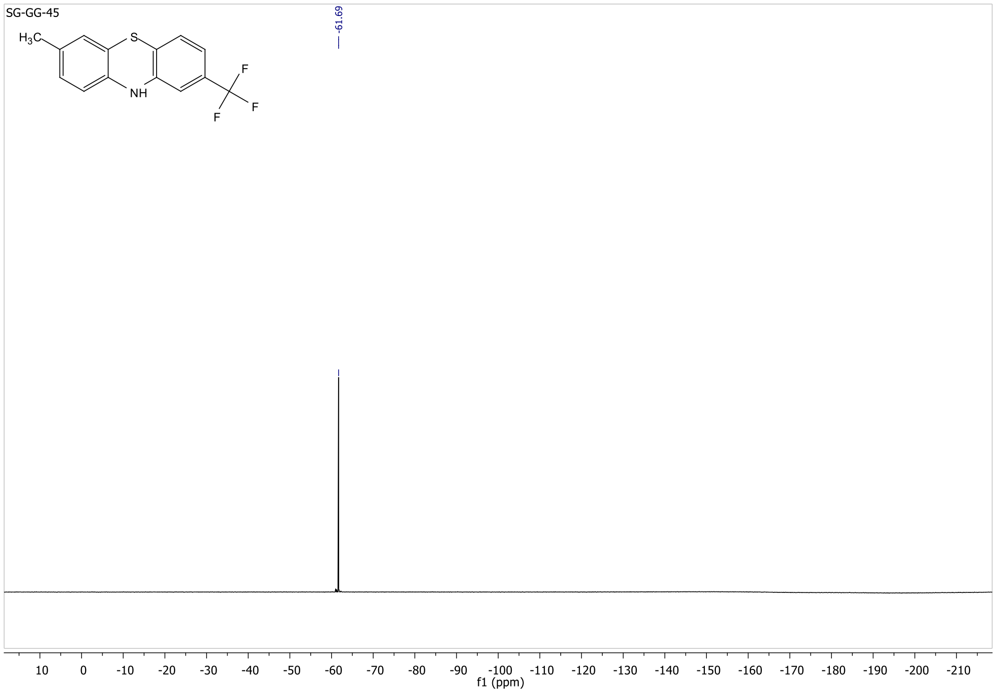

Figure S13. ^{19}F NMR of **2k** in DMSO-d_6.

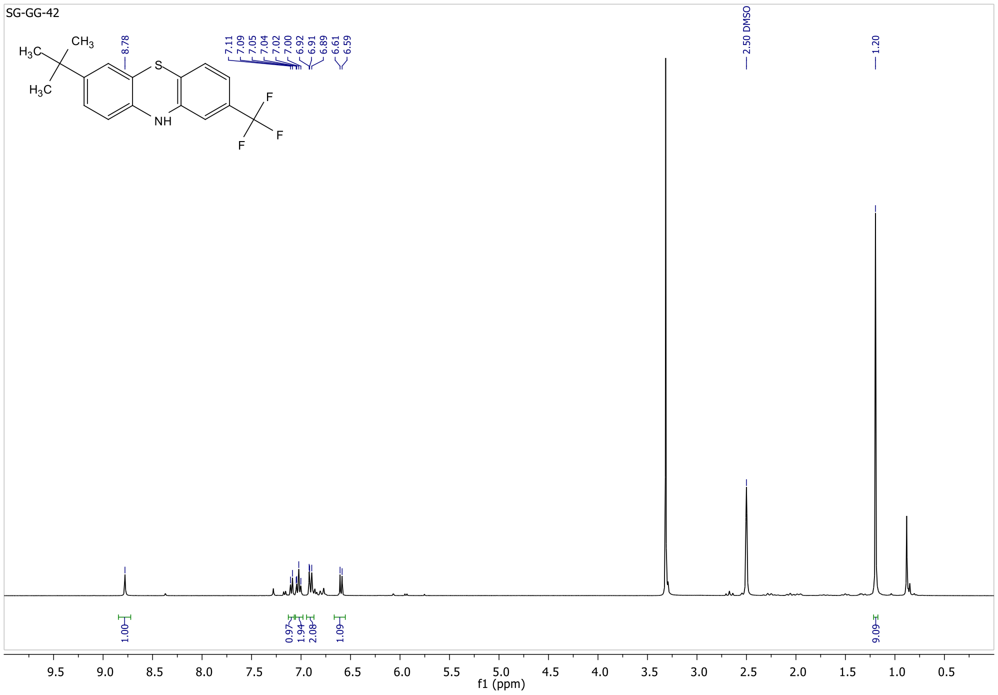

Figure S14. ^{1}H NMR of **2l** in DMSO-d_6.

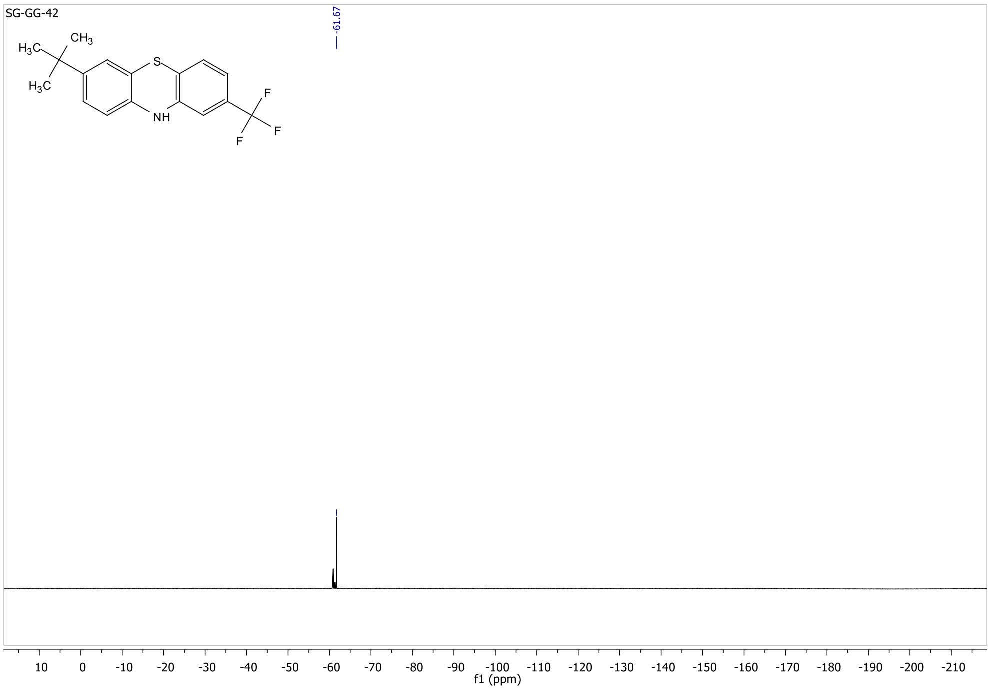

Figure S15. ^{19}F NMR of **2l** in DMSO-d_6.

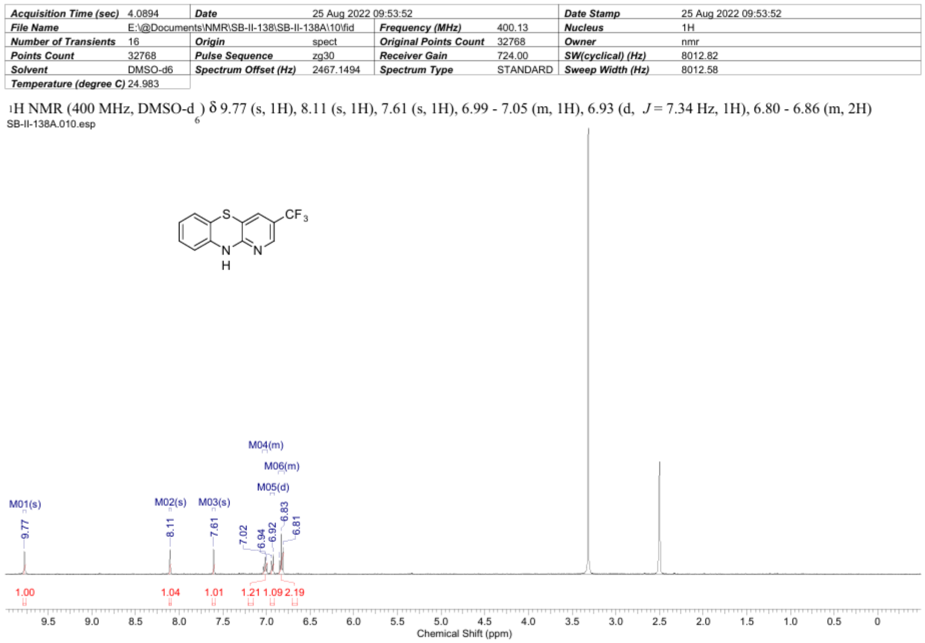

Figure S16. ^{1}H NMR of **2n** in DMSO-d_6.

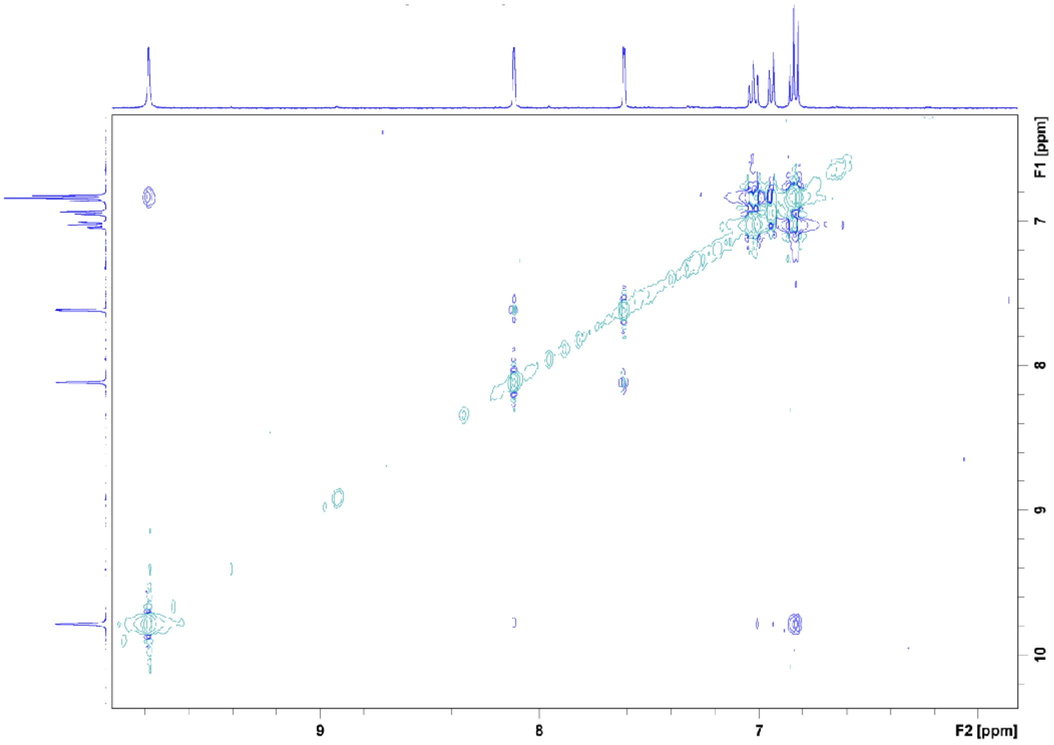

Figure S17. NOESY NMR of **2n** in DMSO-d_6.

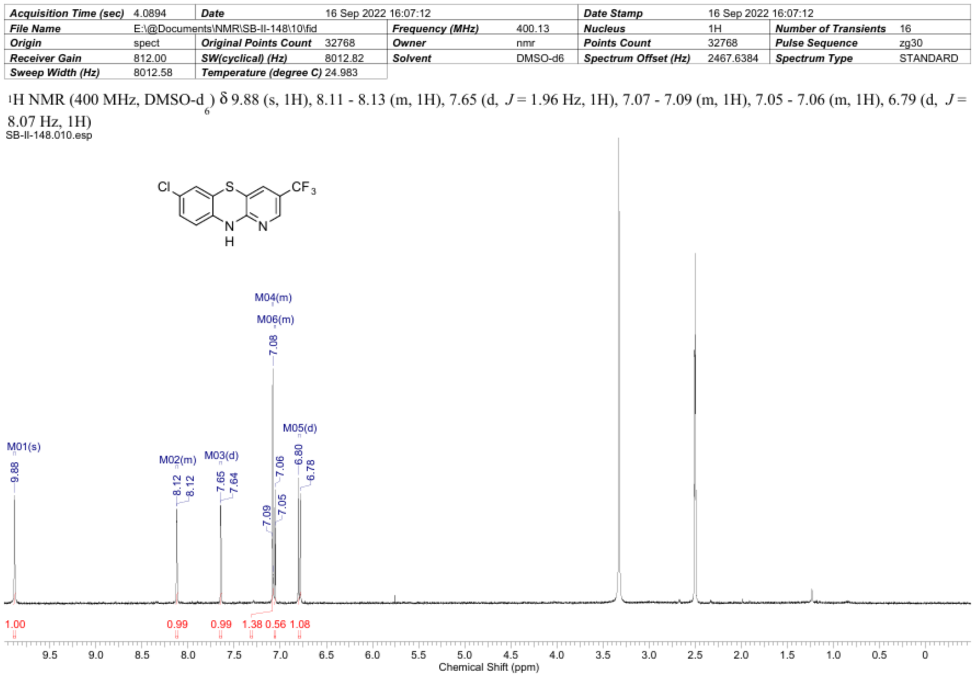

Figure S18. ^{1}H NMR of **2o** in DMSO-d_6.

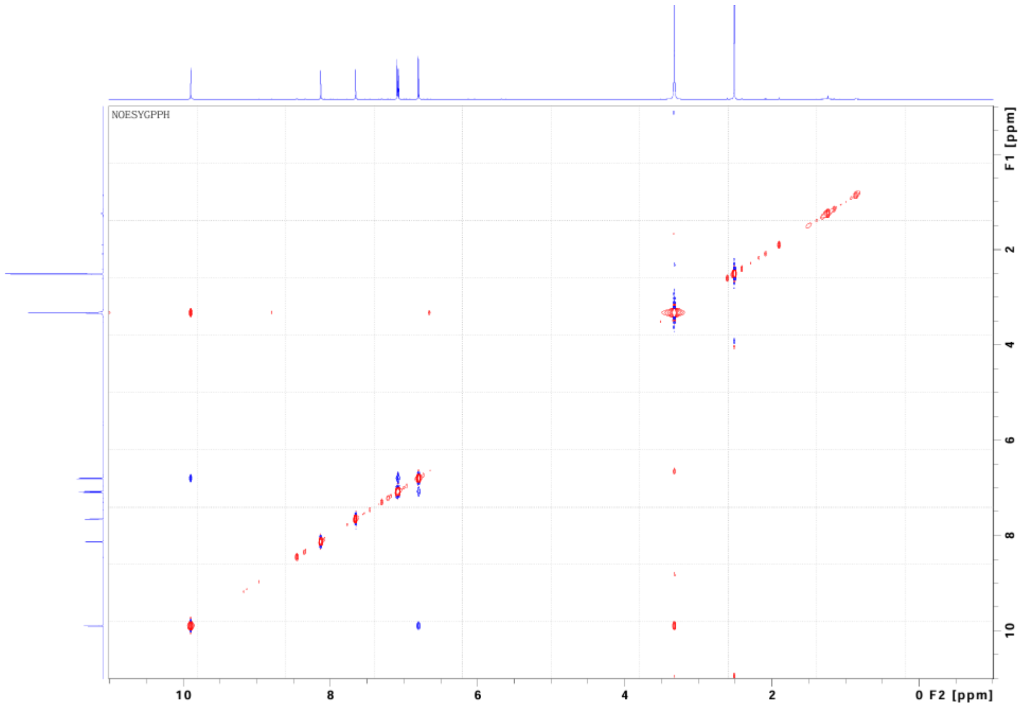

Figure S19. NOESY NMR of **2o** in DMSO-d_6.

**Acquisition Time (sec)**	4.0894	**Comment**	CK-04	**Date**	03 Jul 2020 13:44:16		
**Date Stamp**	03 Jul 2020 13:44:16			**File Name**	\\Mac\Dropbox\ Meyers Lab Data\Carlos Kamal Lab Data\NMR Data\CK-04\10\fid		
**Frequency (MHz)**	400.13	**Nucleus**	1H	**Number of Transients**	16	**Origin**	spect
**Original Points Count**	32768	**Owner**	nmr	**Points Count**	32768	**Pulse Sequence**	zg30
**Receiver Gain**	724.00	**SW(cyclical) (Hz)**	8012.82	**Solvent**	METHANOL-d4		
**Spectrum Offset (Hz)**	2463.3376	**Spectrum Type**	STANDARD	**Sweep Width (Hz)**	8012.58	**Temperature (degree C)**	25.091

^{1}H NMR (400 MHz, METHANOL-d_4) δ 7.50 - 7.58 (m, 4H), 7.10 (d, *J* = 7.34 Hz, 2H), 7.01 (br. s., 2H), 6.88 (br. s., 2H), 6.77 (d, *J* = 6.36 Hz, 2H), 5.18 (br. s., 2H), 4.42 (s, 2H), 3.91 (d, *J* = 3.18 Hz, 2H), 3.61 (br. s., 8H), 3.40 (br. s., 2H)

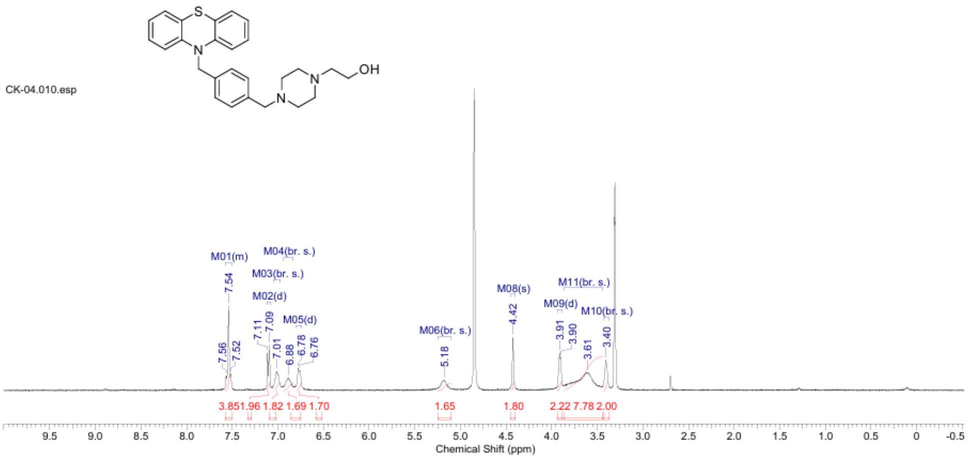

Figure S20. ^{1}H NMR of **21a** in Methanol-d_4.

**Acquisition Time (sec)**	4.0894	**Comment**	CK-06	**Date**	03 Jul 2020 13:20:48		
**Date Stamp**	03 Jul 2020 13:20:48			**File Name**	\\Mac\Dropbox\ Meyers Lab Data\Carlos Kamal Lab Data\NMR Data\CK-06\10\fid		
**Frequency (MHz)**	400.13	**Nucleus**	1H	**Number of Transients**	16	**Origin**	spect
**Original Points Count**	32768	**Owner**	nmr	**Points Count**	32768	**Pulse Sequence**	zg30
**Receiver Gain**	724.00	**SW(cyclical) (Hz)**	8012.82	**Solvent**	METHANOL-d4		
**Spectrum Offset (Hz)**	2463.8264	**Spectrum Type**	STANDARD	**Sweep Width (Hz)**	8012.58	**Temperature (degree C)**	24.983

^{1}H NMR (400 MHz, METHANOL-d_4) δ 7.59 (d, *J* = 8.07 Hz, 2H), 7.47 (d, *J* = 7.82 Hz, 2H), 6.67 (br. s., 6H), 6.39 (br. s., 2H), 4.89 - 5.14 (m, 2H), 4.44 (s, 2H), 3.89 - 3.94 (m, 2H), 3.47 - 3.86 (m, 8H), 3.41 (br. s., 2H)

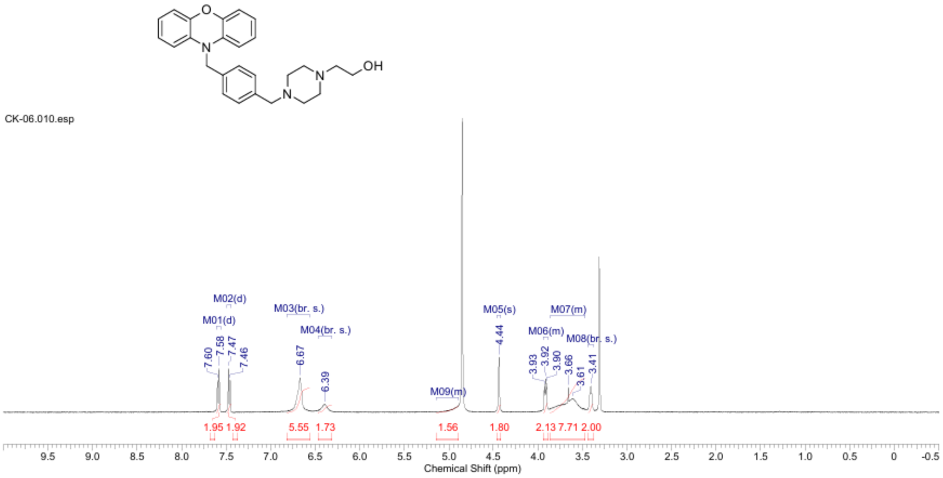

Figure S21. ^{1}H NMR of **21b** in Methanol-d_4.

Acquisition Time (sec)	4.0894	Comment	CK-08	Date	03 Jul 2020 13:25:04		
Date Stamp	03 Jul 2020 13:25:04			File Name	\\Mac\Dropbox\ Meyers Lab Data\Carlos Kamal Lab Data\NMR Data\CK-08\10\fid		
Frequency (MHz)	400.13	Nucleus	1H	Number of Transients	16	Origin	spect
Original Points Count	32768	Owner	nmr	Points Count	32768	Pulse Sequence	zg30
Receiver Gain	575.00	SW(cyclical) (Hz)	8012.82	Solvent	METHANOL-d4		
Spectrum Offset (Hz)	2465.2937	Spectrum Type	STANDARD	Sweep Width (Hz)	8012.58	Temperature (degree C)	25.091

^{1}H NMR (400 MHz, METHANOL-d_4) δ 8.12 (dd, J = 2.08, 7.70 Hz, 2H), 7.43 - 7.48 (m, 4H), 7.37 - 7.43 (m, 2H), 7.23 - 7.27 (m, 2H), 7.19 - 7.23 (m, 2H), 5.64 (d, J = 2.45 Hz, 2H), 4.26 (br. s., 2H), 3.84 - 3.89 (m, 2H), 3.61 (br. s., 4H), 3.41 (br. s., 4H), 3.32 - 3.36 (m, 2H)

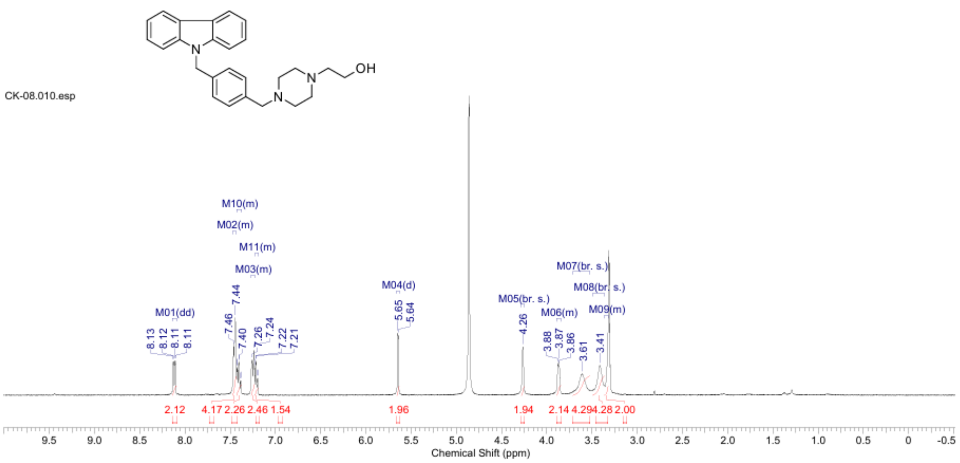

Figure S22. ^{1}H NMR of **21c** in Methanol-d_4.

Acquisition Time (sec)	4.0894	Comment	CK-12	Date	03 Jul 2020 13:35:44		
Date Stamp	03 Jul 2020 13:35:44			File Name	\\Mac\Dropbox\ Meyers Lab Data\Carlos Kamal Lab Data\NMR Data\CK-12\10\fid		
Frequency (MHz)	400.13	Nucleus	1H	Number of Transients	16	Origin	spect
Original Points Count	32768	Owner	nmr	Points Count	32768	Pulse Sequence	zg30
Receiver Gain	575.00	SW(cyclical) (Hz)	8012.82	Solvent	METHANOL-d4		
Spectrum Offset (Hz)	2463.5820	Spectrum Type	STANDARD	Sweep Width (Hz)	8012.58	Temperature (degree C)	24.983

^{1}H NMR (400 MHz, METHANOL-d_4) δ 7.44 - 7.55 (m, 4H), 7.17 - 7.25 (m, 4H), 7.00 - 7.07 (m, 4H), 6.87 - 6.94 (m, 2H), 5.05 (s, 2H), 4.30 (br. s., 2H), 3.89 (dd, J = 4.16, 5.87 Hz, 2H), 3.37 - 3.85 (m, 8H), 3.34 (br. s., 2H)

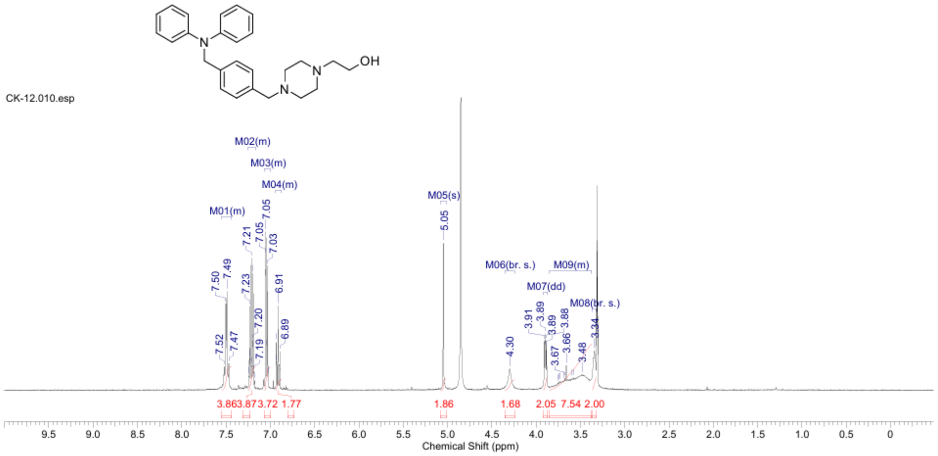

Figure S23. ^{1}H NMR of **21d** in Methanol-d_4.

Acquisition Time (sec)	4.0894	Comment	CK-14	Date	03 Jul 2020 13:40:00		
Date Stamp	03 Jul 2020 13:40:00			File Name	\\Mac\Dropbox_Meyers Lab Data\Carlos Kamal Lab Data\NMR Data\CK-14\10\fid		
Frequency (MHz)	400.13	Nucleus	1H	Number of Transients	16	Origin	spect
Original Points Count	32768	Owner	nmr	Points Count	32768	Pulse Sequence	zg30
Receiver Gain	645.00	SW(cyclical) (Hz)	8012.82	Solvent	METHANOL-d4		
Spectrum Offset (Hz)	2463.8264	Spectrum Type	STANDARD	Sweep Width (Hz)	8012.58	Temperature (degree C)	25.091

^{1}H NMR (400 MHz, METHANOL-d_4) δ 7.58 (d, *J* = 7.82 Hz, 2H), 7.48 (d, *J* = 8.07 Hz, 2H), 7.14 (d, *J* = 7.82 Hz, 2H), 7.07 (dd, *J* = 1.47, 7.58 Hz, 2H), 7.03 (dt, *J* = 1.47, 7.70 Hz, 2H), 6.86 (dt, *J* = 0.98, 7.34 Hz, 2H), 5.00 (s, 2H), 4.36 (s, 2H), 3.88 - 3.92 (m, 2H), 3.48 - 3.80 (m, 8H), 3.36 - 3.41 (m, 2H), 3.22 (s, 4H)

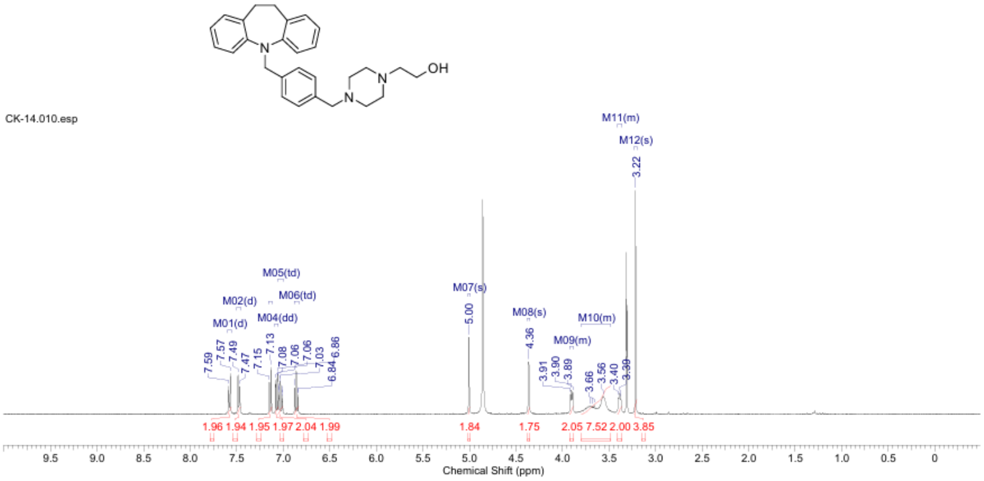

Figure S24. ^{1}H NMR of **21e** in Methanol-d_4.

Acquisition Time (sec)	4.0894	Comment	CK-10	Date	03 Jul 2020 13:29:20				
Date Stamp	03 Jul 2020 13:29:20			File Name	D:\Carlos NMR Data\CK-10\10\fid			Frequency (MHz)	400.13
Nucleus	1H	Number of Transients	16	Origin	spect	Original Points Count	32768	Owner	nmr
Points Count	32768	Pulse Sequence	zg30	Receiver Gain	575.00	SW(cyclical) (Hz)	8012.82	Solvent	METHANOL-d4
Spectrum Offset (Hz)	2464.3154	Spectrum Type	STANDARD			Sweep Width (Hz)	8012.58	Temperature (degree C)	25.091

^{1}H NMR (400 MHz, METHANOL-d_4) δ 7.49 - 7.59 (m, 4H), 7.11 (d, *J* = 7.58 Hz, 1H), 7.06 (d, *J* = 8.07 Hz, 1H), 7.01 - 7.04 (m, 1H), 6.87 - 6.94 (m, 2H), 6.79 (d, *J* = 7.82 Hz, 1H), 6.74 (d, *J* = 1.71 Hz, 1H), 5.16 (br. s., 2H), 4.39 (br. s., 2H), 3.91 (br. s., 2H), 3.47 - 3.79 (m, 8H), 3.39 (br. s., 2H)

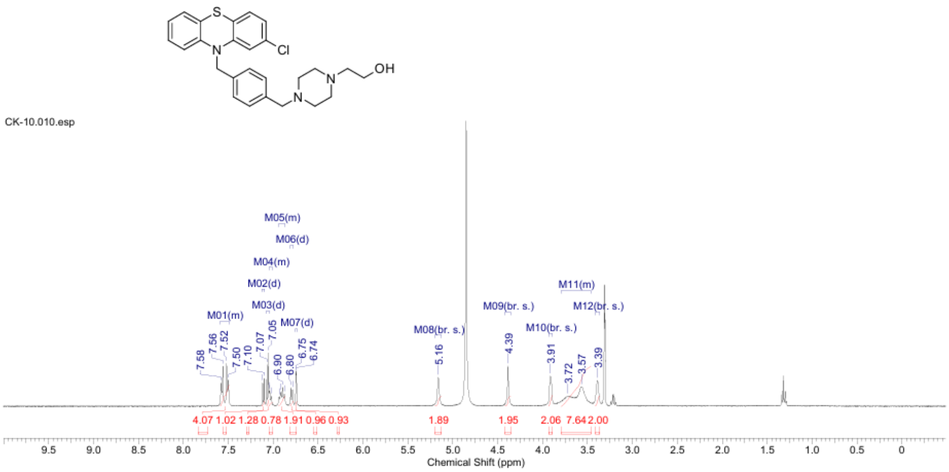

Figure S25. ^{1}H NMR of **21f** in Methanol-d_4.

Acquisition Time (sec)	4.0894	Date	08 Nov 2021 10:14:56			Date Stamp	08 Nov 2021 10:14:56		
File Name	H:\@Documents\NMR\SB-II-77\10\fid			Frequency (MHz)	400.13	Nucleus	1H	Number of Transients	16
Origin	spect	Original Points Count	32768	Owner	nmr	Points Count	32768	Pulse Sequence	zg30
Receiver Gain	575.00	SW(cyclical) (Hz)	8012.82	Solvent	DMSO-d6	Spectrum Offset (Hz)	2467.3940	Spectrum Type	STANDARD
Sweep Width (Hz)	8012.58	Temperature (degree C)	21.965						

^{1}H NMR (400 MHz, DMSO-d_6) δ 7.57 (br. s., 2H), 7.40 (d, *J* = 7.34 Hz, 2H), 7.35 (s, 2H), 7.13 - 7.19 (m, 2H), 7.11 (t, *J* = 7.34 Hz, 1H), 6.93 - 6.99 (m, 1H), 6.82 (d, *J* = 8.31 Hz, 1H), 5.21 (s, 2H), 3.76 (br. s., 2H), 3.69 (dd, *J* = 5.26, 14.31 Hz, 2H), 3.48 (dd, *J* = 4.40, 11.25 Hz, 8H), 3.20 (br. s., 2H)

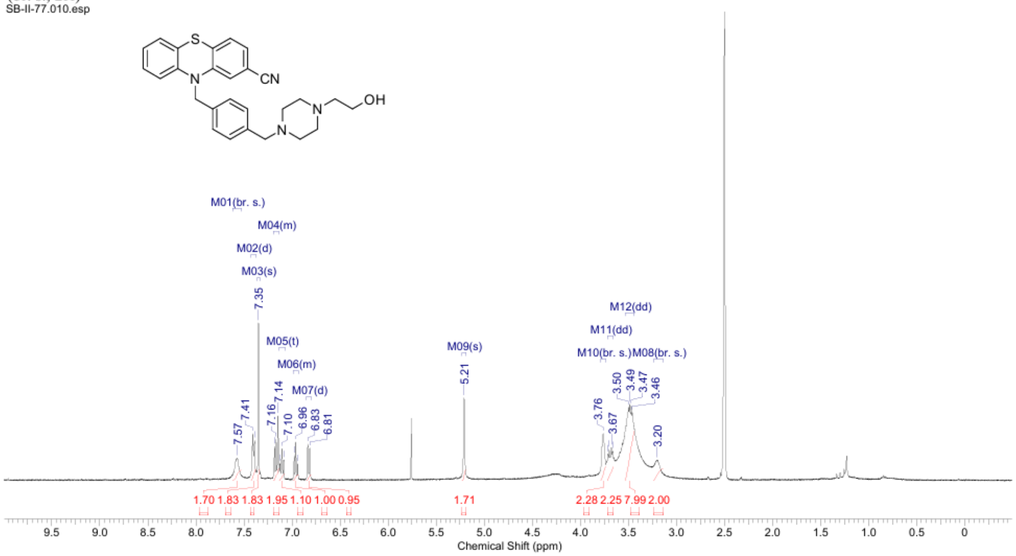

Figure S26. ^{1}H NMR of **21g** in DMSO-d_6.

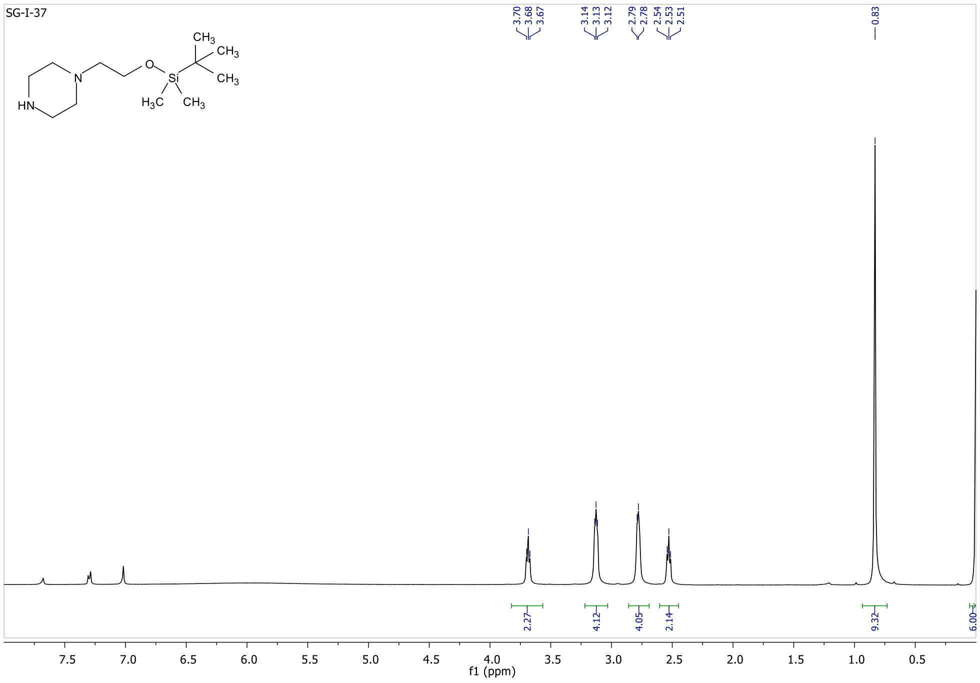

Figure S27. ^{1}H NMR of **22** in $CDCl_3$.

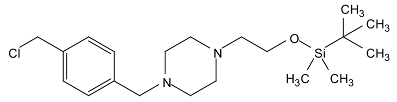

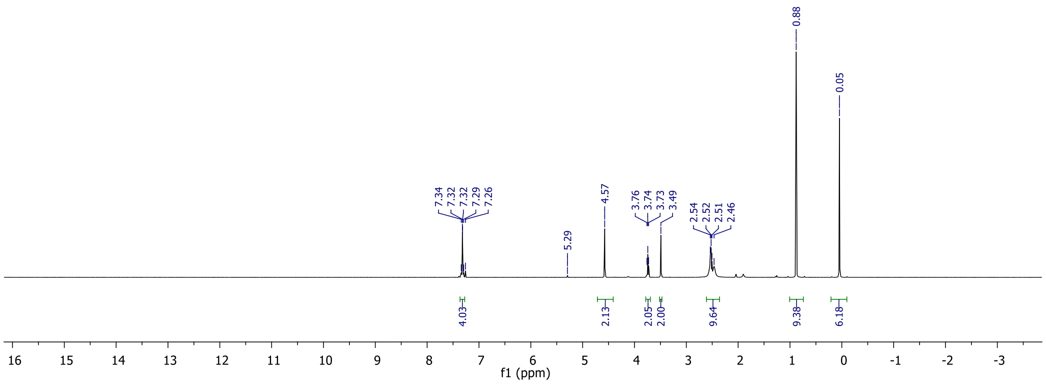

Figure S28. ^{1}H NMR of **24** in $CDCl_3$.

Acquisition Time (sec)	4.0894	***Date***	16 Jun 2022 11:02:08			***Date Stamp***	16 Jun 2022 11:02:08		
File Name	E:\@Documents\NMR\SB-II-122\10\fid			***Frequency (MHz)***	400.13	***Nucleus***	1H	***Number of Transients***	16
Origin	spect	***Original Points Count***	32768	***Owner***	nmr	***Points Count***	32768	***Pulse Sequence***	zg30
Receiver Gain	724.00	***SW(cyclical) (Hz)***	8012.82	***Solvent***	DMSO-d6	***Spectrum Offset (Hz)***	2467.8831	***Spectrum Type***	STANDARD
Sweep Width (Hz)	8012.58	***Temperature (degree C)***	25.091						

^{1}H NMR (400 MHz, DMSO-d_6) δ 7.25 (d, *J* = 5.38 Hz, 4H), 7.11 - 7.15 (m, 1H), 7.06 (s, 1H), 7.02 (d, *J* = 8.56 Hz, 1H), 6.90 (s, 1H), 6.80 (d, *J* = 8.31 Hz, 1H), 6.52 (s, 1H), 6.35 (d, *J* = 2.45 Hz, 1H), 5.10 (s, 2H), 4.30 - 4.37 (m, 1H), 3.59 (s, 3H), 3.46 (d, *J* = 5.87 Hz, 2H), 3.39 (s, 2H), 2.35 (br. s., 8H)

SB-II-122.010.esp

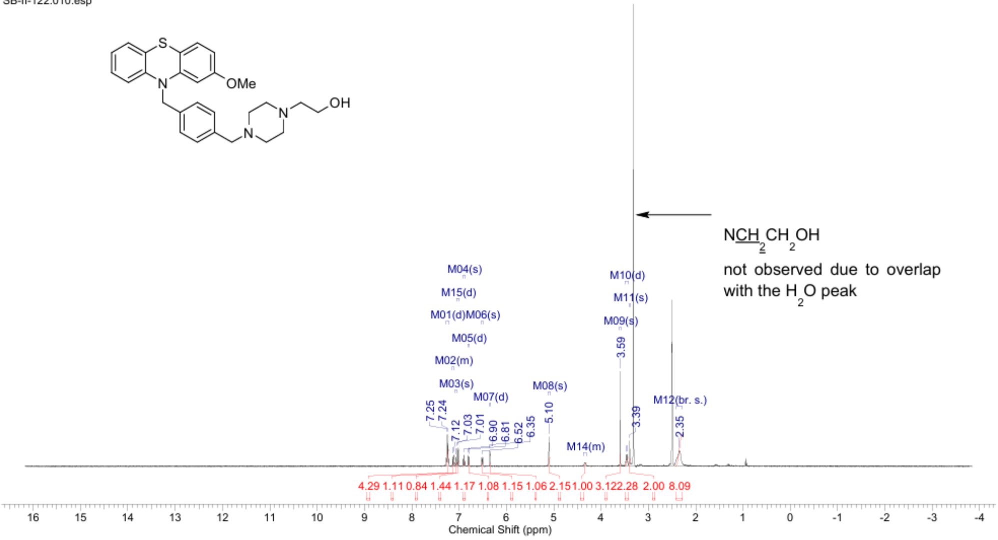

Figure S29. ^{1}H NMR of **21h** in DMSO-d_6.

Acquisition Time (sec)	4.0894	Date	10 Jun 2022 11:04:16		Date Stamp	10 Jun 2022 11:04:16		
File Name	E:\@Documents\NMR\SB-II-120\10\fid		Frequency (MHz)	400.13	Nucleus	1H	Number of Transients	16
Origin	spect	Original Points Count	32768	Owner	nmr	Points Count	32768	Pulse Sequence	zg30
Receiver Gain	406.00	SW(cyclical) (Hz)	8012.82	Solvent	DMSO-d6	Spectrum Offset (Hz)	2467.6384	Spectrum Type	STANDARD
Sweep Width (Hz)	8012.58	Temperature (degree C)	25.091					

^{1}H NMR (400 MHz, DMSO-d_6) δ 7.22 (br. s., 4H), 7.11 (d, J = 7.58 Hz, 1H), 7.04 (t, J = 7.58 Hz, 1H), 6.94 (br. s., 1H), 6.86 (dd, J = 7.58, 13.94 Hz, 2H), 6.73 (d, J = 7.83 Hz, 1H), 6.64 (d, J = 8.07 Hz, 1H), 5.06 (br. s., 2H), 4.34 (br. s., 1H), 3.45 (br. s., 2H), 3.38 (br. s., 2H), 2.52 - 2.56 (m, 2H), 2.27 - 2.41 (m, 8H), 2.15 (s, 3H)

SB-II-120.010.esp

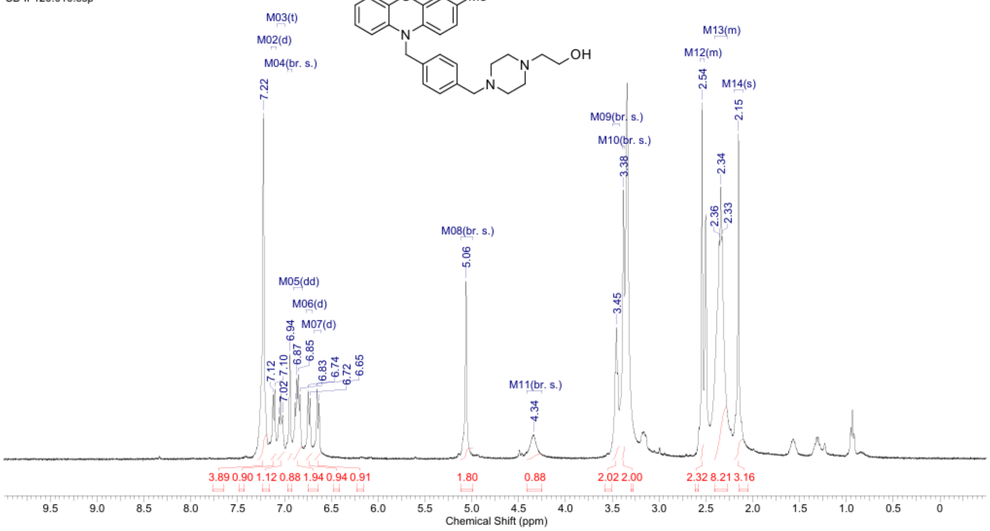

Figure S30. ^{1}H NMR of **21i** in DMSO-d_6.

Acquisition Time (sec)	4.0894	Date	01 Sep 2022 10:28:00		Date Stamp	01 Sep 2022 10:28:00		
File Name	E:\@Documents\NMR\SB-II-139\10\fid		Frequency (MHz)	400.13	Nucleus	1H	Number of Transients	16
Origin	spect	Original Points Count	32768	Owner	nmr	Points Count	32768	Pulse Sequence	zg30
Receiver Gain	362.00	SW(cyclical) (Hz)	8012.82	Solvent	DMSO-d6	Spectrum Offset (Hz)	2450.5212	Spectrum Type	STANDARD
Sweep Width (Hz)	8012.58	Temperature (degree C)	24.983					

^{1}H NMR (400 MHz, DMSO-d_6) δ 7.42 (s, 1H), 7.36 (d, J = 8.56 Hz, 1H), 7.21 (s, 4H), 7.11 (d, J = 7.34 Hz, 1H), 7.02 - 7.08 (m, 1H), 6.88 - 6.94 (m, 1H), 6.82 (d, J = 8.56 Hz, 1H), 6.76 (d, J = 8.31 Hz, 1H), 5.12 (s, 2H), 3.45 (t, J = 6.11 Hz, 2H), 3.37 (s, 2H), 2.45 - 2.47 (m, 2H), 2.28 - 2.43 (m, 8H)

SB-II-139.010.esp

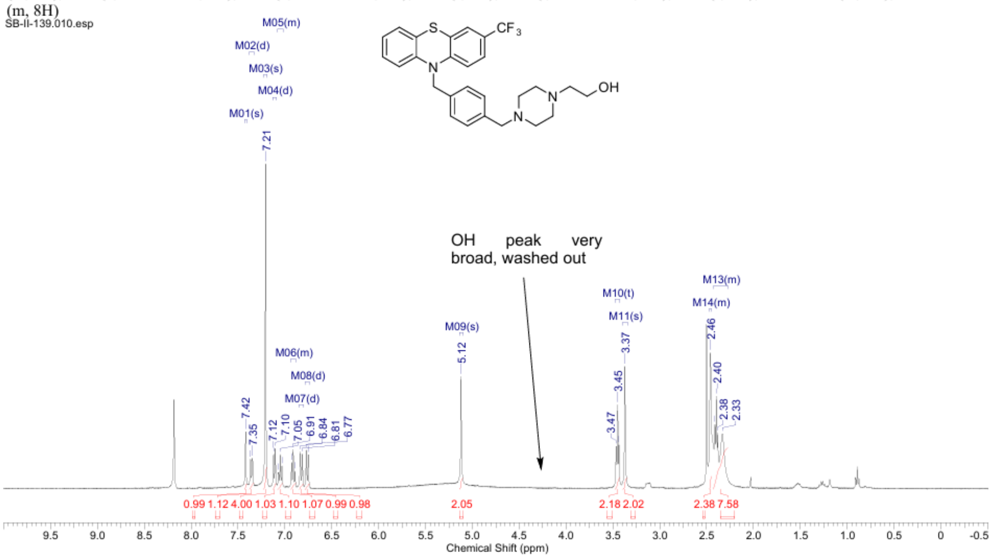

Figure S31. ^{1}H NMR of **21j** in DMSO-d_6.

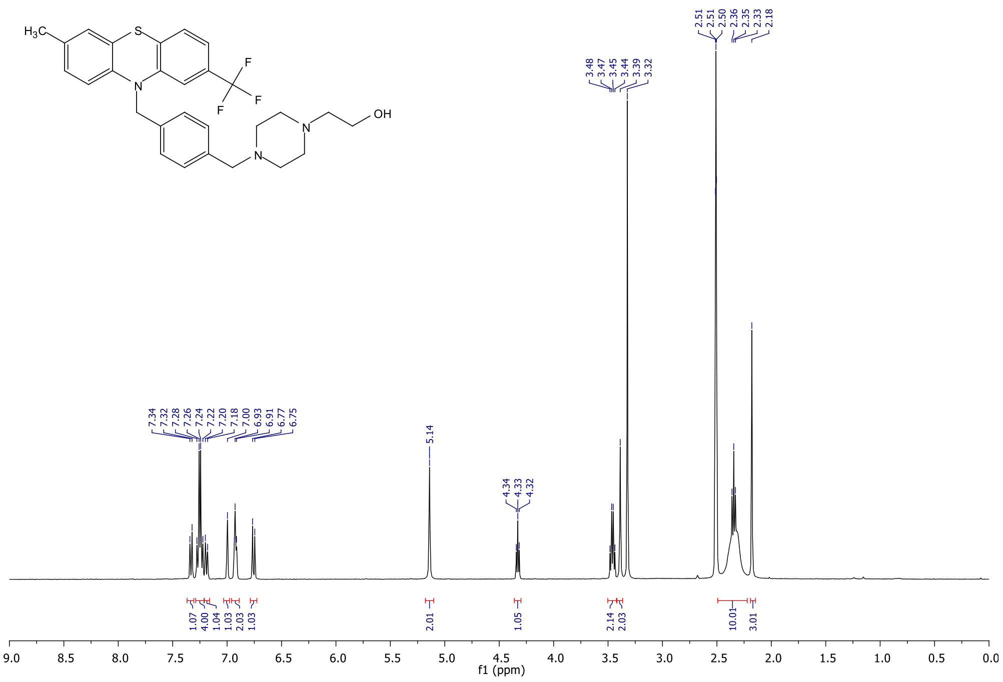

Figure S32. ^{1}H NMR of **21k** in DMSO-d_6.

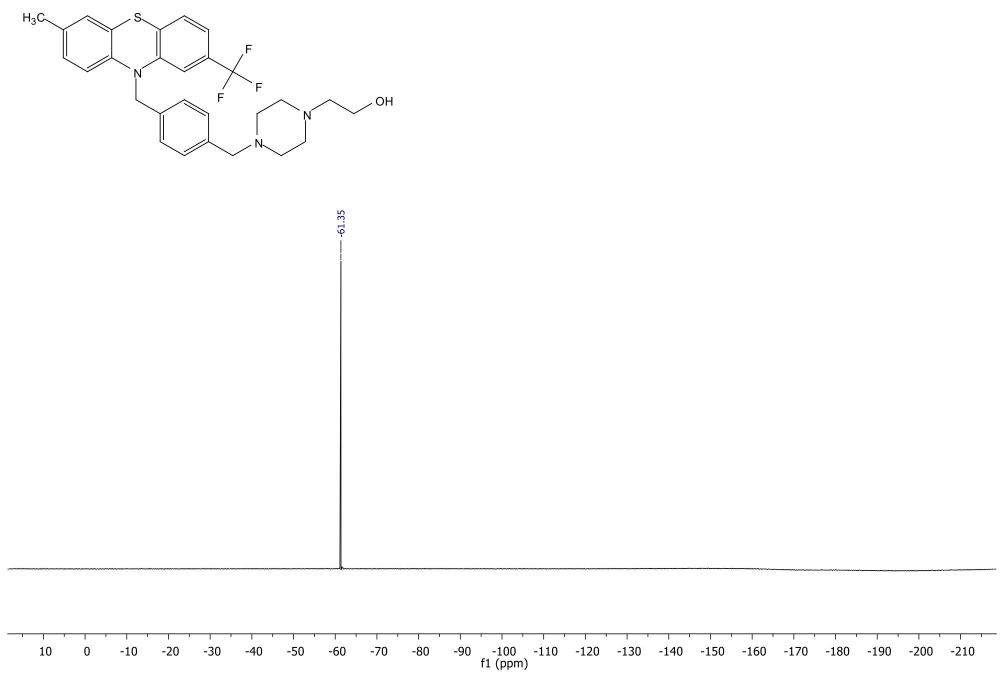

Figure S33. ^{19}F NMR of **21k** in DMSO-d_6.

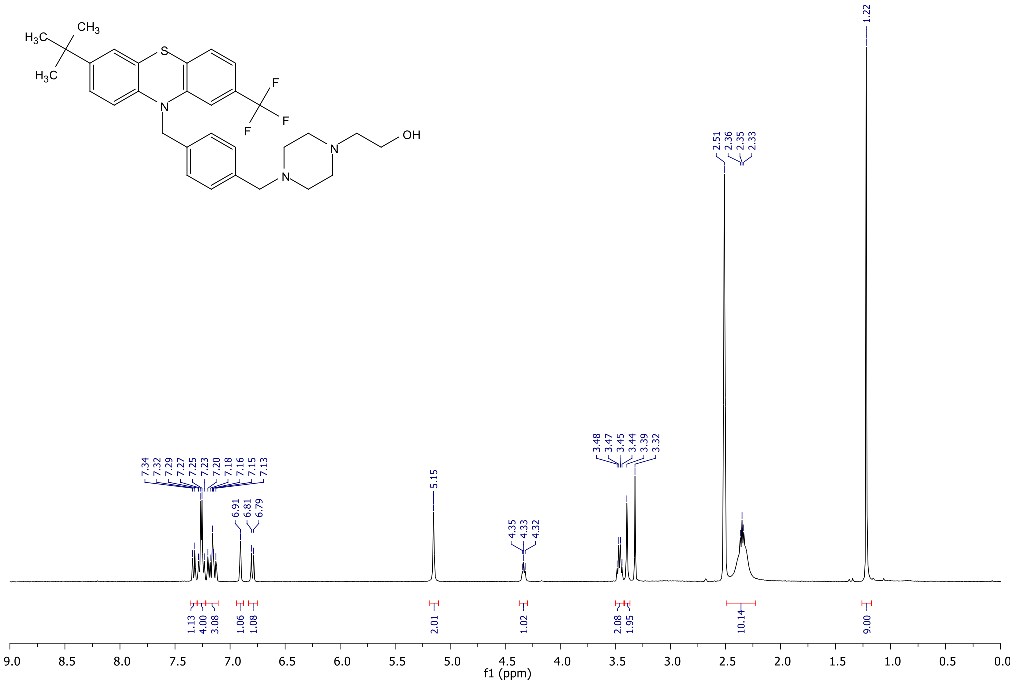

Figure S34. ^{1}H NMR of **21l** in DMSO-d_6.

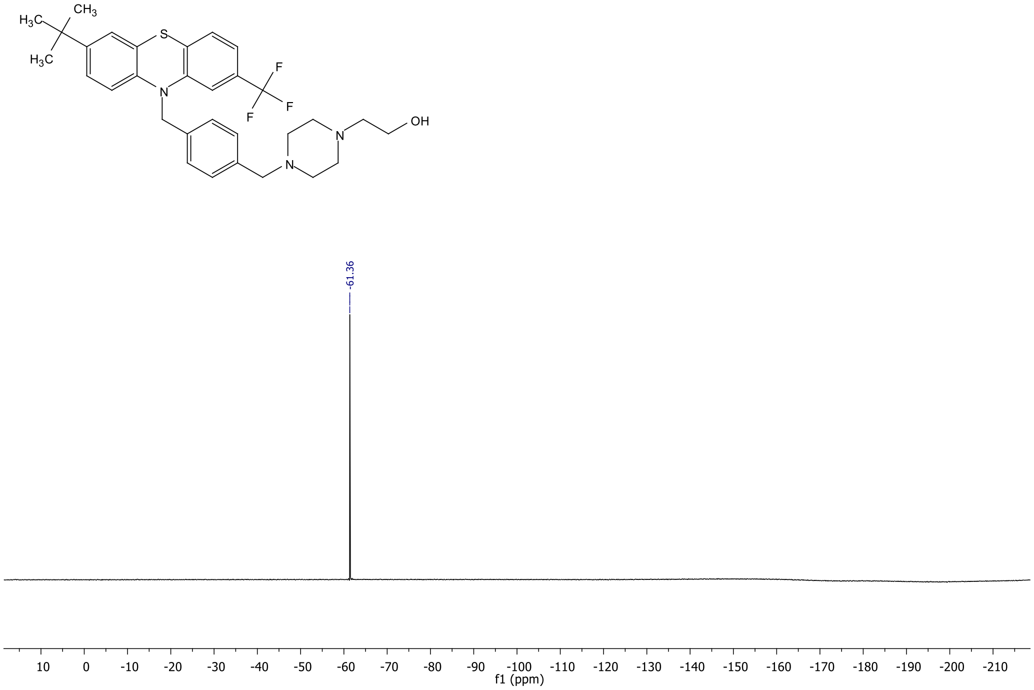

Figure S35. ^{19}F NMR of **21l** in DMSO-d_6.

Acquisition Time (sec)	4.0894	Date	07 Jul 2022 11:19:12			Date Stamp	07 Jul 2022 11:19:12		
File Name	E:\@Documents\NMR\SB-II-129\10\fid			Frequency (MHz)	400.13	Nucleus	1H	Number of Transients	16
Origin	spect	Original Points Count	32768	Owner	nmr	Points Count	32768	Pulse Sequence	zg30
Receiver Gain	362.00	SW(cyclical) (Hz)	8012.82	Solvent	DMSO-d6	Spectrum Offset (Hz)	2466.9050	Spectrum Type	STANDARD
Sweep Width (Hz)	8012.58	Temperature (degree C)	24.983						

^{1}H NMR (400 MHz, DMSO-d_6) δ 7.94 (dd, *J* = 1.22, 4.89 Hz, 1H), 7.46 (dd, *J* = 1.22, 7.58 Hz, 1H), 7.13 - 7.26 (m, 4H), 7.08 (d, *J* = 6.60 Hz, 1H), 6.98 - 7.03 (m, 1H), 6.85 - 6.90 (m, 2H), 6.72 (d, *J* = 8.31 Hz, 1H), 5.30 (s, 2H), 3.51 (br. s., 2H), 3.46 (t, *J* = 6.24 Hz, 2H), 3.38 (s, 2H), 2.36 (t, *J* = 6.36 Hz, 8H)

SB-II-129.010.esp

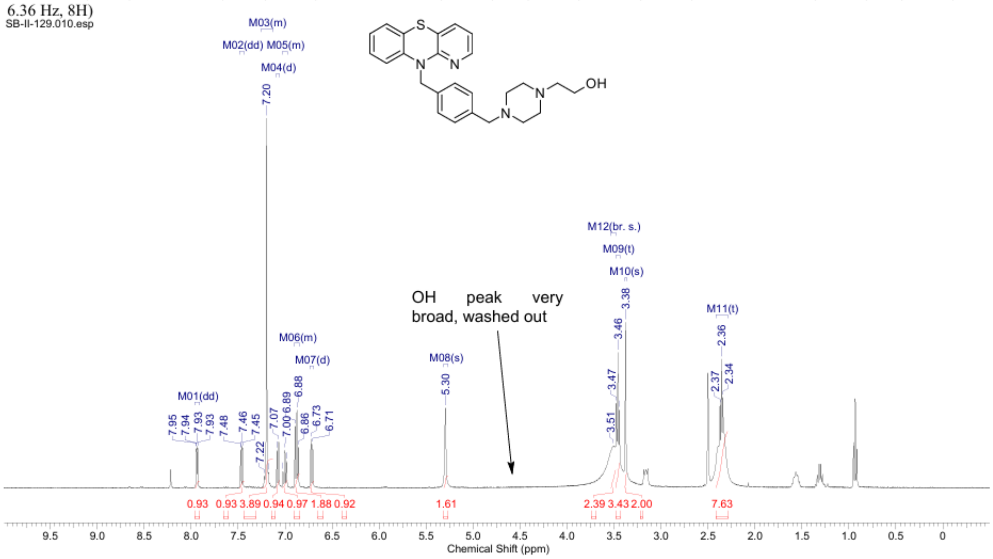

Figure S36. ^{1}H NMR of **21m** in DMSO-d_6.

Acquisition Time (sec)	4.0894	Date	01 Sep 2022 10:30:08			Date Stamp	01 Sep 2022 10:30:08		
File Name	E:\@Documents\NMR\SB-II-140\10\fid			Frequency (MHz)	400.13	Nucleus	1H	Number of Transients	16
Origin	spect	Original Points Count	32768	Owner	nmr	Points Count	32768	Pulse Sequence	zg30
Receiver Gain	512.00	SW(cyclical) (Hz)	8012.82	Solvent	DMSO-d6	Spectrum Offset (Hz)	2466.4158	Spectrum Type	STANDARD
Sweep Width (Hz)	8012.58	Temperature (degree C)	24.983						

^{1}H NMR (400 MHz, DMSO-d_6) δ 8.26 (s, 1H), 7.82 (s, 1H), 7.17 - 7.23 (m, 4H), 7.10 (d, *J* = 7.34 Hz, 1H), 7.01 - 7.07 (m, 1H), 6.91 - 6.96 (m, 1H), 6.75 (d, *J* = 8.07 Hz, 1H), 5.33 (br. s., 2H), 4.38 (br. s, 1H), 3.46 (t, *J* = 6.24 Hz, 2H), 3.38 (s, 2H), 2.27 - 2.43 (m, 8H)

SB-II-140.010.esp

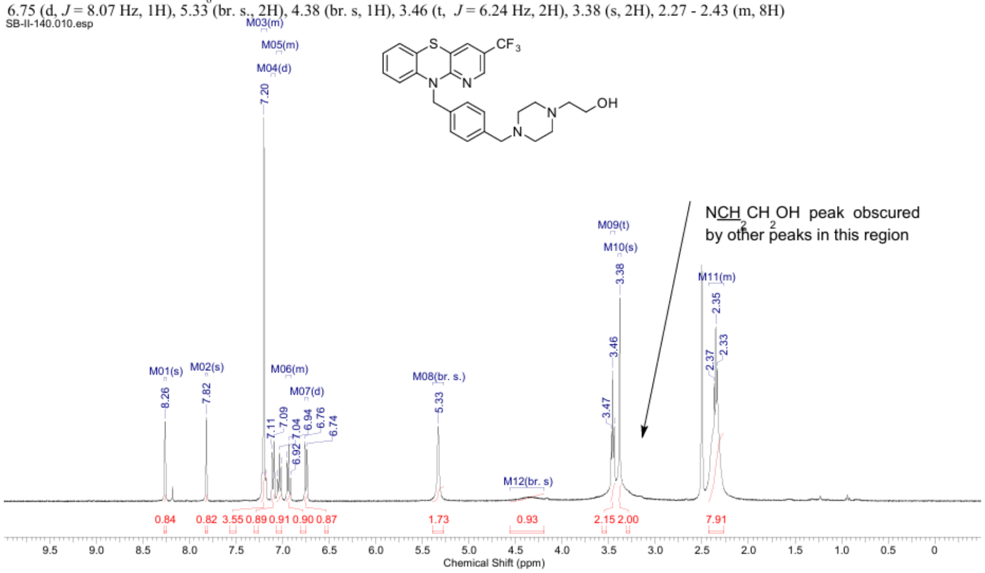

Figure S37. ^{1}H NMR of **21n** in DMSO-d_6.

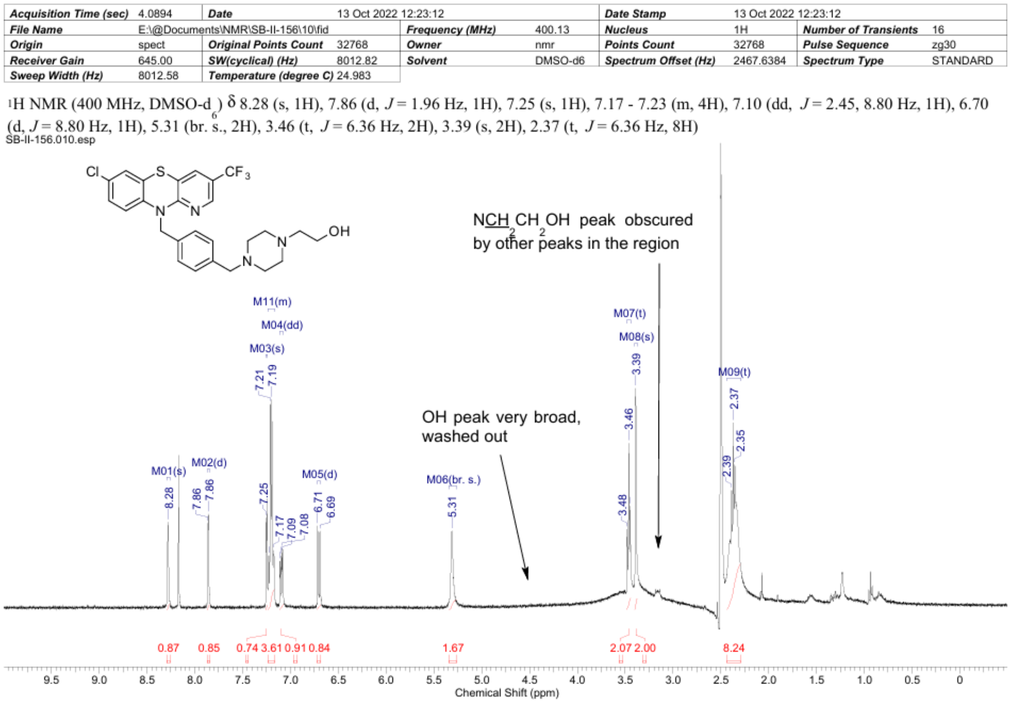

Figure S38. ^{1}H NMR of **21o** in DMSO-d_6.
